# Liver-targeted degradation of BRD4 reverses hepatic fibrosis and enhances metabolism in murine models

**DOI:** 10.7150/thno.113852

**Published:** 2025-06-18

**Authors:** Shengjie Yuan, Ayesha Nisar, Chuanjie Chen, Xin Dong, Yongzhang Pan, Meiting Zi, Qiong Wang, Sawar Khan, Yaxun Guo, Xuan Zhang, Yonghan He

**Affiliations:** 1 State Key Laboratory of Genetic Evolution & Animal Models, Key Laboratory of Healthy Aging Research of Yunnan Province, Kunming Institute of Zoology, Chinese Academy of Sciences, Kunming, Yunnan, China.; 2 Drug Discovery & Development Center, Shanghai Institute of Materia Medica, Chinese Academy of Sciences, Shanghai, China.; 3 National Resource Center for Non-Human Primates, Kunming Institute of Zoology, Chinese Academy of Sciences, Kunming, Yunnan, China.; 4 Department of Cell Biology, School of Life Sciences, Central South University, Changsha, Hunan, China.; 5 Institute of Molecular Biology and Biotechnology, The University of Lahore, Lahore, Pakistan.; 6 Department of Breast Surgery, The Second Hospital of Shandong University, Jinan, Shandong, China.; 7 University of Chinese Academy of Sciences, Beijing, China.

**Keywords:** asialoglycoprotein receptor, BRD4 degradation, liver fibrosis, liver-targeting chimera, proteolysis-targeting chimera

## Abstract

**Background:** Liver fibrosis, characterized by excessive extracellular matrix deposition, is a precursor to cirrhosis and hepatocellular carcinoma, and current treatments are often limited by off-target toxicities.

**Methods and results:** We repurposed the liver-targeting chimera (LIVTAC) XZ1606, a novel proteolysis-targeting chimera (PROTAC) conjugated with a triantennary *N*-acetylgalactosamine (tri-GalNAc) moiety, to degrade BRD4 in hepatic stellate cells. *In vitro*, XZ1606 induced potent, dose- and time-dependent BRD4 degradation in LX-2 cells via the ubiquitin-proteasomal pathway after ASGPR-mediated endocytosis, with minimal cytotoxicity in normal hepatocytes. TGF-β-activated LX-2 cells exhibited significant reductions in fibrotic markers upon treatment, correlating with decreased BRD4 levels. *In vivo*, XZ1606 (1.5 mg/kg) significantly ameliorated fibrosis in both CCl₄-induced and choline-deficient L-amino acid-defined high-fat diet (CDAA-HFD) mouse models, as evidenced by reduced collagen deposition and normalized transcriptomic and metabolomic profiles. Notably, key proinflammatory and profibrotic genes and metabolites, including 1-Methylnicotinamide, were downregulated.

**Conclusion:** These results highlight the therapeutic potential of LIVTAC XZ1606 in reversing liver fibrosis and steatosis through targeted BRD4 degradation, offering a novel and selective approach for chronic liver disease treatment.

## Introduction

Liver fibrosis, a dynamic wound-healing response to chronic liver injury, is a major global health burden that can ultimately progress to cirrhosis and hepatocellular carcinoma (HCC) 1, 2. The pathogenesis of liver fibrosis involves the activation of hepatic stellate cells (HSCs), which transdifferentiate into myofibroblast-like cells and secrete excessive extracellular matrix (ECM) proteins 3-5. This progressive accumulation of ECM disrupts the normal liver architecture and function, ultimately leading to liver failure. Despite considerable advances in understanding the molecular pathways driving fibrogenesis, current therapeutic strategies remain limited and are often associated with significant off-target toxicities 6, 7. Current therapeutic strategies for liver fibrosis primarily focus on treating the underlying causes, such as antiviral agents for viral hepatitis 8, lifestyle interventions for alcohol-related and metabolic liver diseases 9, and metabolic modulators for nonalcoholic steatohepatitis (NASH) 10. Pharmacological candidates aiming to directly target fibrotic mechanisms include angiotensin receptor blockers (ARBs) 11, peroxisome proliferator-activated receptor (PPAR) agonists 12, and inhibitors of key signaling pathways such as transforming growth factor beta (TGF-β) 13 and platelet-derived growth factor (PDGF) 14. Additionally, monoclonal antibodies like simtuzumab 15 and small molecules targeting integrins or lysyl oxidase like 2 (LOXL2) 16 have been tested in clinical trials. However, many of these approaches have shown limited efficacy or have been discontinued due to off-target effects or lack of clinical benefit. These challenges underscore the urgent need for innovative, selective, and tissue-specific therapies that can effectively disrupt the fibrotic cascade with minimal systemic toxicity. Thus, there is an urgent need for novel, targeted approaches that can selectively modulate the fibrotic process without compromising systemic safety.

At the molecular level, the activation of HSCs is driven by a complex interplay of cytokines and growth factors, with TGF-β playing a central role in initiating and perpetuating fibrogenic signaling 17-19. TGF-β not only stimulates ECM production but also modulates the expression of various genes involved in inflammation and cellular proliferation 20-22. Among the downstream mediators, the bromodomain-containing protein BRD4 has emerged as a key regulator of transcriptional programs that drive fibrosis 23-27. BRD4 facilitates the assembly of transcriptional complexes on acetylated histones, thereby promoting the expression of pro-fibrotic and pro-inflammatory genes 28-30. Although small-molecule inhibitors targeting BRD4 have shown promise in preclinical models, their clinical translation has been hampered by dose-limiting toxicities due to off-target effects in non-hepatic tissues 29, 31. The emergence of proteolysis-targeting chimeras (PROTACs) has revolutionized the field of targeted protein degradation 32, 33. PROTACs are heterobifunctional molecules that harness the cellular ubiquitin-proteasome system to selectively degrade target proteins 30, 34, 35. Unlike traditional inhibitors, which require sustained occupancy of their targets, PROTACs act catalytically, enabling efficient and sustained protein degradation even at low concentrations 36-38. This catalytic mode of action offers significant therapeutic advantages, including overcoming issues of drug resistance that often arise with conventional inhibitors 39. However, systemic administration of PROTACs may result in undesirable degradation of proteins in non-target tissues, necessitating strategies to enhance their tissue specificity.

To address this challenge, liver-targeted delivery systems have been developed to direct therapeutic agents specifically to hepatocytes and HSCs 40. One promising strategy exploits the asialoglycoprotein receptor (ASGPR), which is highly expressed on the surface of liver cells. Conjugation of therapeutic agents to a triantennary *N*-acetylgalactosamine (GalNAc) moiety enables selective binding to ASGPR and facilitates receptor-mediated endocytosis 40. This targeted approach has been successfully employed in the delivery of siRNAs and antisense oligonucleotides for liver diseases, and it holds great promise for the targeted delivery of PROTACs as well. We have developed a Liver-Targeting Chimera (LIVTAC) approach using a PROTAC molecule coupled to ASGPR through an innovative linker, and successfully applied it to HCC treatment 40.

In this context, we repurposed the LIVTAC XZ1606, designed to selectively degrade BRD4 in HSCs. Upon binding to ASGPR, XZ1606 is internalized into HSCs where the cleavable linker is processed by cathepsin B, releasing the active PROTAC. This leads to selective degradation of BRD4 and the subsequent downregulation of fibrotic gene expression. By specifically targeting BRD4, XZ1606 aims to mitigate the aberrant activation of HSCs and interrupt the progression of liver fibrosis. We hypothesized that targeted degradation of BRD4 via XZ1606 would not only attenuate the fibrotic response *in vitro* but also ameliorate hepatic fibrosis and steatosis *in vivo*. To test this hypothesis, we performed a comprehensive series of experiments in both *in vitro* and *in vivo* models of liver fibrosis. The transcriptomic and metabolomic analyses revealed that XZ1606 treatment reversed the pro-fibrotic gene expression and metabolic disturbances characteristic of these disease states. The development of LIVTAC XZ1606 represents a significant advancement in the field of targeted protein degradation, offering a novel and selective approach for the treatment of liver fibrosis. By combining the high specificity of ASGPR-mediated targeting with the catalytic efficiency of PROTAC-mediated degradation, XZ1606 provides a promising therapeutic strategy to address the unmet clinical need in chronic liver diseases.

## Materials and Methods

### Cell culture and maintenance

LX-2 human HSCs and AML12 mouse hepatocytes were purchased from American Type Culture Collection (ATCC, Manassas, VA, USA) and cultured in Dulbecco's Modified Eagle Medium (DMEM; C11995500BT, Gibco, MD, USA) supplemented with 10% fetal bovine serum (FBS; RY-F22, Royacel Biotechnology Co., Ltd., Lanzhou, China) and 1% penicillin/streptomycin (15140-122, Gibco, MD, USA). Cells were maintained in a humidified incubator at 37 °C with 5% CO₂. Routine testing for mycoplasma contamination was performed to ensure culture integrity.

### Cell viability assay

Cell viability was quantified using the CellTiter-Glo Luminescent Cell Viability Assay Kit (G1111, Promega, Madison, WI, USA). LX-2 cells were seeded in 96-well plates at a density of 4 × 10³ cells per well in 100 µL of complete DMEM. After 24 h, the medium was replaced with 200 µL of DMEM containing various concentrations of the compounds under investigation. Cells were incubated for an additional 24, 48, or 72 h at 37 °C before absorbance was measured following the manufacturer's protocol. Dose-response curves were generated, and IC₅₀ values were calculated using GraphPad Prism v8 (San Diego, CA, USA).

### Fibroblast activation assay

To induce a fibrotic phenotype, LX-2 cells were seeded in 6-well plates and allowed to adhere for 12 h. The medium was then replaced with DMEM containing 0.5% FBS for serum starvation over 12 h. Fibrosis was induced by treating the cells with TGF-β1 (5 and 10 ng/mL; 10804-HNAC, Sino Biological Inc., Beijing, China). For treatment groups, XZ1606 was added 12 h post-TGF-β1 stimulation and maintained for 16 h. Cells were subsequently harvested for protein analysis by Western blot.

### Generation of ASGPR-knockdown cells

To evaluate the expression of ASGPR in liver tissue, publicly available data from the Human Protein Atlas (THPA) database was utilized. ASGPR knockdown in LX-2 cells was achieved using short hairpin RNA (shRNA) constructs. Two shRNA sequences targeting ASGPR (shRNA-1: 5'-GCAATGTGGGAAGAAAGAT-3'; shRNA-2: 5'-GCACCACATAGGCCCTGTGAA-3') were cloned into the pLKO.1 lentiviral vector. HEK-293T cells were transfected with the lentiviral vectors using polyethyleneimine (HY-K2014, MedChemExpress, Shanghai, China) at a 3:2:1 mass ratio to produce viral particles. Viral supernatants were harvested at 48- and 72-h post-transfection, filtered through 0.45 μm PVDF membranes, and then used to transduce LX-2 cells at 60-70% confluence with 8 μg/mL polybrene. After three days, transduced cells were selected in medium containing 1 μg/mL puromycin (ant-pr-1, InvivoGen, USA). Knockdown efficiency was confirmed via Western blot analysis.

### Western blot analysis

For protein extraction, LX-2 cells were washed with phosphate-buffered saline (PBS) and lysed in ice-cold RIPA buffer (BP 115DG, Boston BioProducts, Ashland, MA, USA) supplemented with EDTA and EGTA. Cell lysates were collected after scraping and centrifugation at 13,000 rpm for 15 min at 4 °C. For mouse liver tissues, samples were harvested, snap-frozen in liquid nitrogen, and homogenized in 200 µL of RIPA buffer using a tissue homogenizer (KZ-III-FP, SERvicebio, Wuhan, China). Lysates were incubated on ice for 30 min and centrifuged as described, and protein concentrations were determined using a bicinchoninic acid (BCA) protein assay kit (P0010, Beyotime, Shanghai, China).

Equal amounts of protein were mixed with 4× Laemmli sample buffer (1610747, Bio-Rad, Hercules, CA, USA) containing 5% β-mercaptoethanol and heated at 95 °C for 8 min. Proteins were resolved on 10% SDS-PAGE gels and transferred to 0.22-μm polyvinylidene fluoride (PVDF) membranes (Millipore, Bedford, MA, USA) using a wet transfer system (Bio-Rad, Richmond, CA, USA). Membranes were blocked in 5% (w/v) skim milk in tris-buffered saline with 0.1% Tween-20 (TBS-T) for 1 h at room temperature, followed by overnight incubation at 4 °C with primary antibodies (listed in **Table S1**). After washing, membranes were incubated with appropriate secondary antibodies for 1 h at room temperature. Protein bands were visualized using the GeneGnome XRQ-NPC System (Synoptics, Frederick, MD) or SCG-W3000 Plus (Servicebio, Wuhan, China), and quantified with ImageJ software.

### Quantitative Real-Time PCR (qRT-PCR)

Total RNA was extracted from cells and liver tissues using TRIzol reagent (15596018, Invitrogen, CA, USA) per the manufacturer's instructions. RNA quality and quantity were assessed before reverse transcription into cDNA using the RevertAid First-Strand cDNA Synthesis Kit (K1622, Thermo Fisher Scientific, MA, USA). qRT-PCR was performed using 2× Tsingke® Master qPCR Mix (TSE201, Tsingke, Beijing, China) on a CFX-Connect real-time PCR system (Bio-Rad, Richmond, CA, USA), with actin used as the endogenous control. Relative gene expression was calculated using the 2^^-ΔΔCt^ method, and primer sequences are provided in **Table S2**.

### Ubiquitination assay

Following plasmid transfection into LX-2 cells for 48 h, the cells were treated with XZ1606 (100 nM) for 8 h. To inhibit proteasomal degradation, MG-132 (20 µM) was added 4 h before sample collection. Cells were harvested and lysed in immunoprecipitation (IP) lysis buffer (P0013, Beyotime) for 1 h, followed by centrifugation at 13,000 rpm for 10 min to collect the supernatant. The supernatant was divided into two aliquots. One aliquot was stored at -80 °C as the input sample, and the other was retained for immunoprecipitation as IP sample. For IP, anti-Flag magnetic beads (P2115, Beyotime) were incubated with the lysate overnight at 4 °C with rotation to capture Flag-BRD4, as per the manufacturer's protocol. On the next day, the beads were immobilized on a magnetic rack, and the supernatant was discarded. The beads were resuspended in 1× SDS-PAGE loading buffer, boiled for 10 min, and centrifuged at 13,000 rpm for 2 min. The resulting supernatant was analyzed by Western blot.

### Liver fibrosis models

#### Animal housing and ethics

Male C57BL/6 mice were purchased from the Animal Center of the Kunming Institute of Zoology (Kunming, China) and acclimated under specific pathogen-free (SPF) conditions with free access to standard food and water. Environmental conditions were maintained at 20-21 °C, 40%-60% relative humidity, with a 12-h light/dark cycle. All animal experiments were approved by the Animal Ethics Committee of the Kunming Institute of Zoology, Chinese Academy of Sciences (IACUC-RE-2024-07-003 and IACUC-RE-2024-09-002) and were conducted in accordance with the Guide for the Care and Use of Laboratory Animals.

#### Carbon tetrachloride (CCl₄)-induced liver fibrosis model

To establish the fibrosis model, 8-week-old male mice were intraperitoneally injected with CCl₄ (1.25 mL/kg body weight, dissolved in corn oil; HY-Y1888, MCE, Shanghai, China) every three days for six weeks. Control mice received corn oil only. In a preliminary study, mice with CCl₄-induced fibrosis were treated with XZ1606 at doses of 0.5 mg/kg and 1 mg/kg (intraperitoneally) every three days for three weeks. However, these doses did not produce significant antifibrotic effects, as assessed by fibrosis markers. Based on these findings, a higher dose of 1.5 mg/kg was selected for subsequent experiments for the main study. One week after the final CCl₄ injection, mice in the treatment group received XZ1606 at 1.5 mg/kg (intraperitoneally, in 100 µL saline) every three days for three weeks. Mice were sacrificed 24 h after the final injection, and serum and liver tissues were collected for analysis.

#### Choline-deficient L-amino acid-defined high-fat diet (CDAA-HFD) model

For an alternative fibrosis model, 8-week-old male mice were fed a choline-deficient, L-amino acid-defined high-fat diet (CDAA-HFD; XTMRCD60, Xietong Pharmaceutical Bio-Engineering, Jiangsu, China) for 10 weeks. Control mice received a standard chow diet. From week 7, mice in the treatment group received intraperitoneal injections of XZ1606 at a dose of 1.5 mg/kg every three days for four weeks. Mice were euthanized 24 h after the final injection, and serum and liver tissues were collected for subsequent analyses.

### Biochemical analysis

Serum levels of alanine transaminase (ALT) and alkaline phosphatase (ALP) were measured using commercial assay kits (C009-2-1 and A059-2-2, respectively, Nanjing Jiancheng Bioengineering Institute, Nanjing, Jangsu, China) following the manufacturers' protocols or by the National Resource Center for Non-Human Primates, Kunming Institute of Zoology.

### Histological staining

#### Masson's trichrome staining

Paraffin-embedded liver sections were baked at 65 °C for 20 min, deparaffinized in xylene (three 8 min immersions), and rehydrated through graded ethanol (100%, 95%, 85%, and 75% for 3 min each). Sections were stained using a Masson's Trichrome Staining Kit (BA4079b, Baso, Zhuhai, China) following the manufacturer's instructions. Briefly, sections were incubated with Weigert's iron hematoxylin, differentiated in 1% hydrochloric acid alcohol, stained with Ponceau Red-Acid Fuchsin, treated with phosphomolybdic acid, counterstained with aniline blue, and finally dehydrated and cleared in ethanol and xylene before mounting with neutral balsam.

#### Oil Red O staining

Frozen liver sections (8 µm) were fixed in 10% formalin for 10 min, washed, and pretreated with 60% ethanol for 20-30 s. Sections were incubated with freshly prepared Oil Red O working solution (diluted 3:2 with distilled water) for 10-15 min in the dark. Excess dye was removed by rinsing with 60% ethanol, followed by washing in distilled water. Nuclei were counterstained with Mayer's hematoxylin, and sections were subsequently mounted and imaged.

#### Sirius red staining

Paraffin sections were baked, deparaffinized, and rehydrated as described for Masson's staining. Sections were stained with hematoxylin for 7 min, rinsed, and then incubated with Sirius red solution (BA4356, Baso, Zhuhai, China) for 15 min. Following staining, sections were briefly rinsed and then dehydrated through graded ethanol and cleared in xylene before imaging.

### RNA sequencing and data analysis

Total RNA from LX-2 cells and mouse liver tissues was extracted using TRIzol reagent. Poly(A)-enriched RNA-seq libraries were prepared using standard protocols and sequenced on the Illumina NovaSeq X Plus platform. The mouse genomic data (mouse GRCm39 build) and gene annotation information were downloaded from the Ensembl database (https://www.ensembl.org/). Clean reads were obtained after adapter trimming with trim_galore and aligned to the *Mus musculus* genome (GRCm39) using STAR v2.7.11a (https://github.com/alexdobin/STAR). SAMtools v1.18 (https://github.com/samtools) was used for file conversion, and gene expression quantification was performed with featureCounts V2.0.6 (https://subread.sourceforge.net/featureCounts.html). Differentially expressed genes (DEGs) were identified using the DESeq2 package (https://bioconductor.org/packages/release/bioc/html/DESeq2.html), applying a Benjamini-Hochberg adjusted p-value cutoff of < 0.01 and |log2FC| ≥ 1. Hierarchical clustering, heatmap visualization, and volcano plot generation were performed using the ClusterGVis (https://github.com/junjunlab/ClusterGVis) and ggplot2 packages in R. Functional enrichment analyses for gene ontology and KEGG pathways were conducted using ShinyGO 0.82.

### Metabolomics analysis

Metabolomics was performed on liver tissues using liquid chromatography-tandem mass spectrometry (LC-MS/MS). Tissue samples (25 mg) were placed in tubes containing beads, followed by the addition of 500 μl of an extraction solution composed of methanol:acetonitrile:water (2:2:1, v/v) supplemented with deuterated internal standards. The mixture was vortexed for 30 s, homogenized at 35 Hz for 4 min, and sonicated in a 4 °C water bath for 5 min; this cycle was repeated three times. Samples were then incubated at -40 °C for 1 h to precipitate proteins and subsequently centrifuged at 12,000 × g for 15 min at 4 °C. The supernatant was carefully transferred to a new glass vial for analysis, and a quality control (QC) sample was prepared by pooling equal aliquots of each supernatant. The LC-MS/MS analyses were performed using an UHPLC system (Vanquish, Thermo Fisher Scientific) equipped with a Waters ACQUITY UPLC BEH Amide column (2.1 mm × 50 mm, 1.7 μm) coupled to an Orbitrap Exploris 120 mass spectrometer (Thermo Fisher Scientific). The mobile phase consisted of solvent A (25 mmol/L ammonium acetate and 25 mmol/L ammonia hydroxide in water, pH 9.75) and solvent B (acetonitrile). The auto-sampler was maintained at 4 °C, and the injection volume was set to 2 μL. Data acquisition was carried out in information-dependent acquisition (IDA) mode using Xcalibur software. The electrospray ionization (ESI) source was configured with a sheath gas flow rate of 50 Arb, auxiliary gas flow rate of 15 Arb, a capillary temperature of 320 °C, full MS resolution of 60,000, MS/MS resolution of 15,000, collision energies set to 20/30/40, and spray voltages of 3.8 kV in positive mode or -3.4 kV in negative mode.

Raw data were converted to mzXML format using ProteoWizard and processed with an in-house R-based program utilizing XCMS for feature detection, extraction, alignment, and integration. Metabolite identification was achieved using MS-DIAL software in conjunction with the BiotreeDB (V3.0). The resulting dataset, containing feature numbers, sample names, and normalized feature areas, was imported into SIMCA 18.0.1 (Sartorius Stedim Data Analytics AB, Umea, Sweden) for multivariate analysis. Data were scaled and log-transformed to minimize noise and the impact of high variance. Principal component analysis (PCA) was performed to visualize sample distribution and grouping, with a 95% confidence interval used to identify potential outliers. To visualize group separation and identify significantly altered metabolites, supervised orthogonal projections to latent structures-discriminant analysis (OPLS-DA) was applied. A 7-fold cross-validation was performed to determine the R² and Q² values, with Q² intercept values obtained from 200 permutations to assess model robustness and predictive ability. Variables with a variable importance in the projection (VIP) value > 1 and a p-value < 0.05 (Student's t-test) were considered significantly changed metabolites. Pathway enrichment analysis was conducted using databases such as KEGG (http://www.genome.jp/kegg/) and MetaboAnalyst (http://www.metaboanalyst.ca/).

### Statistical analysis

Data are expressed as mean ± standard error of the mean (SEM). Statistical analyses were performed using GraphPad Prism v8. For comparisons between two groups, an unpaired two-tailed t-test was used, with Welch's correction applied if normality assumptions were not met. For comparisons among multiple groups, one-way analysis of variance (ANOVA) followed by Tukey's or Dunnett's post hoc test was employed. A p-value of less than 0.05 was considered statistically significant.

## Results

### ASGPR mediated uptake of LIVTAC

**Figure 1:**
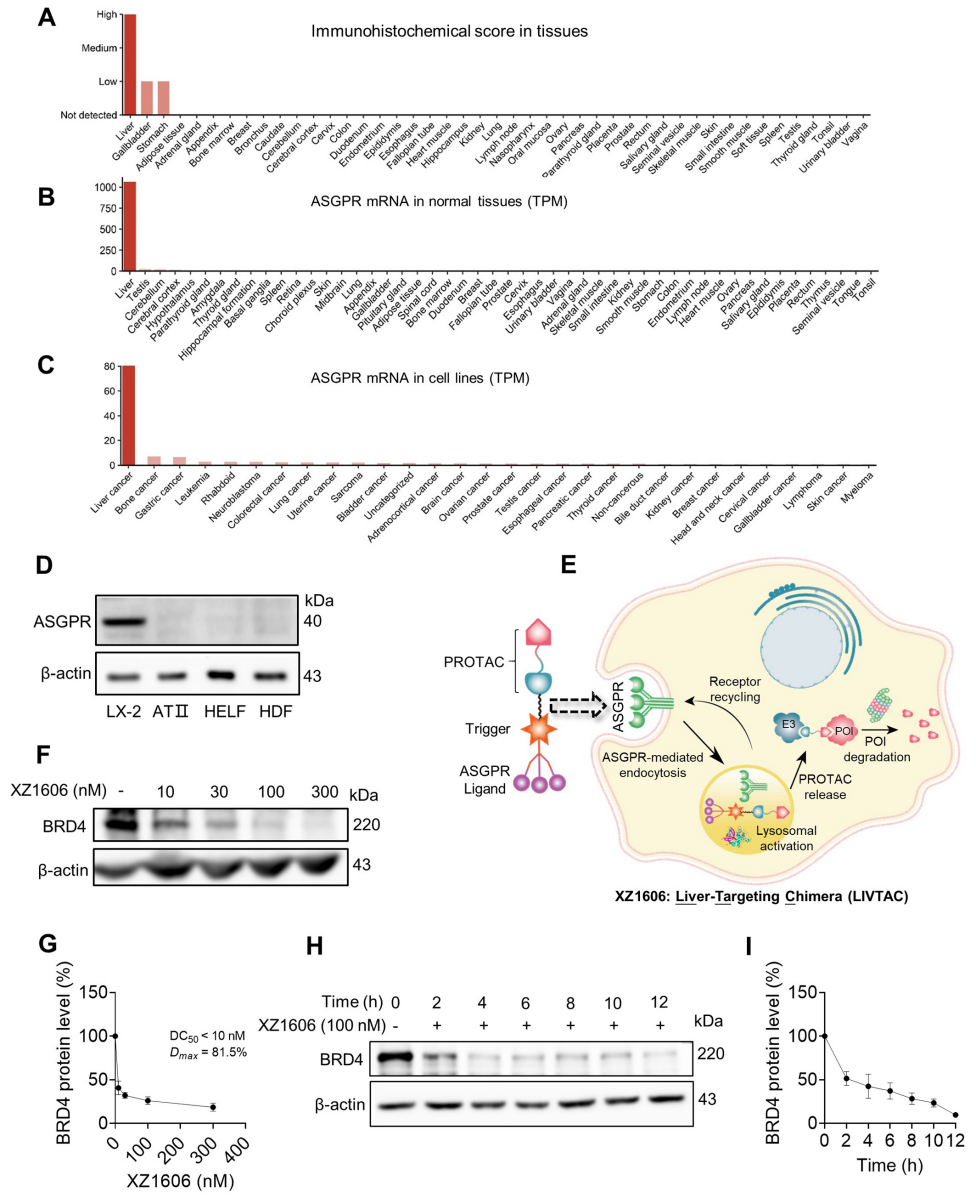
**XZ1606 induces proteasomal degradation of BRD4 in LX-2 cells. (A-C)** ASGPR expression levels in various tissues and cell lines, with liver tissues and cell lines showing the highest expression. Data are retrieved from the Human Protein Atlas (https://www.proteinatlas.org/). **(D)** Western blot analysis showing the absence of ASGPR expression in non-liver-derived cell lines (ATII, HELF, and HDF), confirming ASGPR expression is limited to liver-specific LX-2 cells. **(E)** Schematic representation of the LIVTAC mechanism: The tri-GalNAc moiety selectively binds to the ASGPR receptor on liver cells, facilitating receptor-mediated endocytosis. Upon internalization, the cleavable linker is processed by cathepsin B (CTB), releasing the active PROTAC, which triggers the degradation of the target protein of interest (POI). **(F-G)** Dose-dependent degradation and protein quantification of BRD4 by XZ1606 in LX-2 cells. Western blot analysis shows that treatment with increasing concentrations (10, 30, 100, and 300 nM) of XZ1606 results in a dose-dependent degradation of BRD4. Half-maximal degradation concentration (DC_50_) and maximum level of target degradation (Dmax) were calculated. **(H-I)** Time-dependent degradation and protein quantification of BRD4 by XZ1606 in LX-2 cells. Western blot analysis shows that XZ1606 induces progressive degradation of BRD4 over time (2-12 h), with β-actin as a loading control.

ASGPR is a receptor that plays a crucial role in hepatic endocytosis, facilitating the internalization of glycoproteins containing terminal galactose or *N*-acetylgalactosamine (GalNAc) residues. It is specifically and highly expressed in the liver (Figure 1A), with a transcript per million (TPM) value of 1000, while expression in other tissues is either very low or absent (Figure 1B). Additionally, among various tumor cell lines, ASGPR mRNA expression is most pronounced in liver cancer cell lines (Figure 1C). We further confirmed its specific expression in liver-derived cell lines such as LX-2, while showing very low or no expression in non-liver-derived cell lines, including lung-derived human alveolar type II (ATII), human lung embryo fibroblast (HELF), and skin-derived human dermal fibroblasts (HDF) (Figure 1D).

Leveraging this specificity, we designed XZ1606 to selectively engage ASGPR for enhanced hepatic uptake (Figure 1E), with its exact chemical structure detailed in Figure S1A. XZ1606 was originally developed for HCC 40. Here, we repurposed it as a therapeutic agent against liver fibrosis, which is characterized by excessive extracellular matrix deposition leading to cirrhosis and potentially HCC. Once administered, the tri-GalNAc moiety mediates selective uptake via ASGPR, followed by intracellular cleavage of the linker and release of the active PROTAC. This catalytic degrader binds BRD and recruits an E3 ligase, thereby inducing ubiquitination and proteasomal degradation of BRD protein (Figure 1E).

To evaluate the impact of XZ1606 on HSC viability and its potential selectivity for fibrotic cells over healthy hepatocytes, we performed MTT assays in LX-2 and AML12 cells (Figure S1B-G). In LX-2 cells, XZ1606 displayed minimal cytotoxicity at 24 and 48 h, but at 72 h it reduced viability with an IC₅₀ of 9.408 µM (Figure S1D). In contrast, AML12 cells exhibited negligible changes in viability across all time points, with IC₅₀ values consistently above 10 µM (Figure S1E-G). These data suggest that XZ1606 preferentially affects HSCs while sparing normal hepatocytes, highlighting its potential to alleviate fibrotic processes while mitigating the “on-target off-tissue” effects that commonly limit BRD4 inhibitor therapies.

### LIVTAC degrades BRD4 via a ubiquitin-proteasomal pathway after endocytosis in LX-2 cells

The primary target of LIVTAC in the treatment of liver fibrosis is BRD4, a bromodomain-containing protein known to regulate fibrotic gene expression in HSCs. To assess the efficacy of LIVTAC, we evaluated its ability to degrade BRD4 in LX-2 cells, an HSC line. The mechanistic pathway by which XZ1606 exerts its effects is depicted in the schematic (Figure 1E).

We observed a dose-dependent degradation of BRD4 in LX-2 cells, with increasing concentrations of XZ1606 leading to progressive BRD4 depletion (Figure 1F-G), with half-maximal degradation concentration (DC_50_) at 10 nM and maximum level of target degradation (Dmax) at 81.5% as shown by Western blot quantification (Figure 1G). Time-course analysis revealed that BRD4 degradation is progressive, with maximal degradation occurring within 12 h of XZ1606 treatment (Figure 1H-I). These findings suggest that XZ1606 initiates a dose- and time-dependent degradation process that sustains the loss of BRD4 over an extended period.

**Figure 2:**
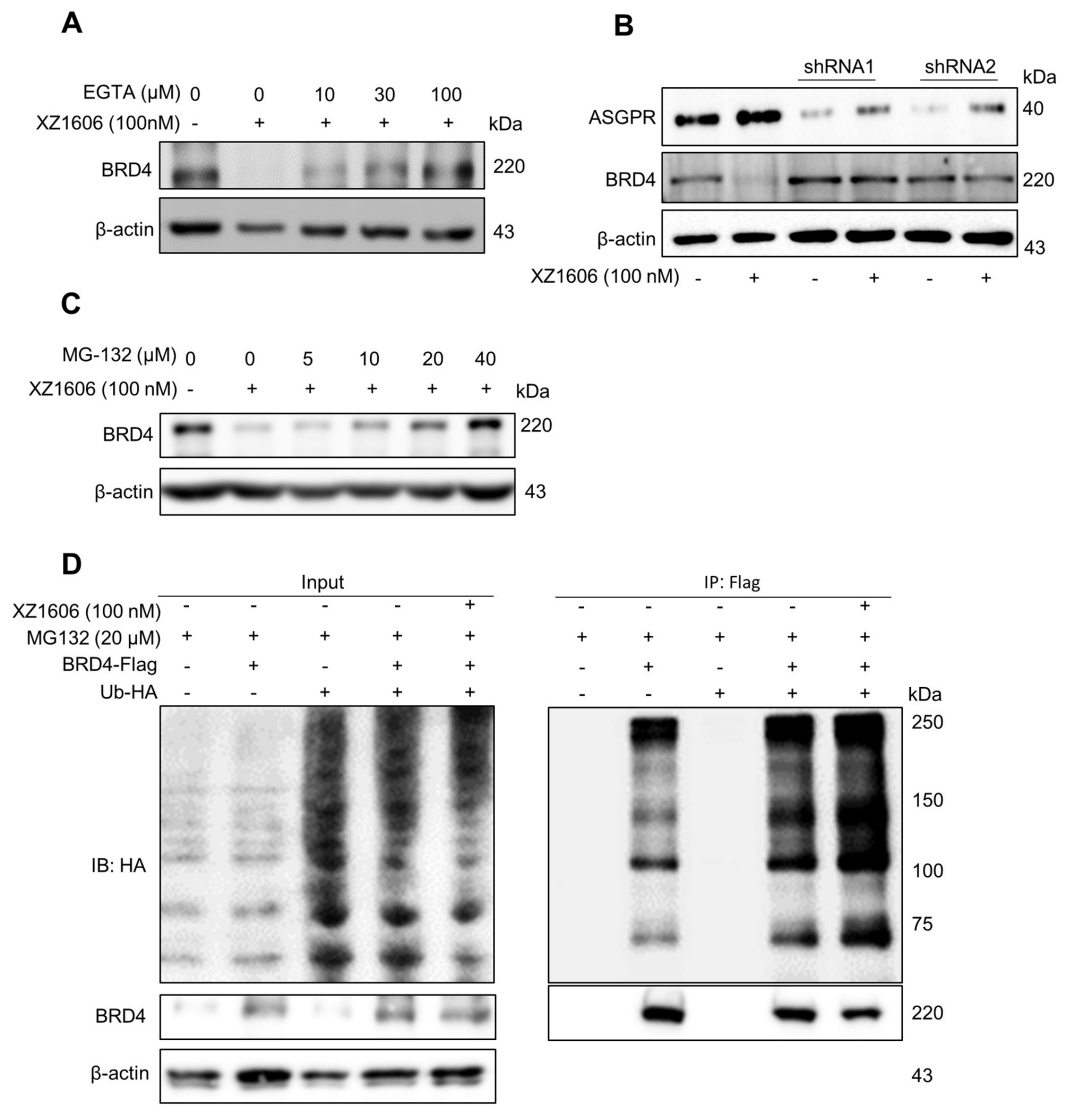
**XZ1606 induces degradation of BRD4 via the ubiquitin-proteasomal pathway in LX-2 cells. (A)** Inhibition of BRD4 degradation by calcium chelation. Pre-treatment with EGTA (calcium chelator) reduces the XZ1606-induced BRD4 degradation, suggesting calcium signaling involvement in the mechanism of action. **(B)** Knockdown of ASGPR blocked the degradation of LIVTAC on BRD4. Small hairpin RNAs (shRNA) were used to knock down the ASGPR in LX-2 cells. **(C)** Proteasomal involvement in BRD4 degradation. Pre-treatment with indicated concentration of MG-132 (proteasomal inhibitor) blocks XZ1606-induced BRD4 degradation.** (D)** Ectopic expression of exogenous BRD4 and ubiquitin. LX-2 cells were treated with 100 nM XZ1606 for 4 h after which 20 μM MG132 were added for an additional 4 h. Cell lysates were collected for Western blot analysis.

To explore the underlying mechanisms further, we assessed the involvement of calcium signaling 41 and the proteasomal pathway 42. Pre-treatment with EGTA, a calcium chelator, significantly reduced the XZ1606-induced degradation of BRD4, indicating that calcium signaling plays a role in the mechanism of action (Figure 2A). Additionally, the degradation of BRD4 by XZ1606 was completely blocked by knockdown of ASGPR (Figure 2B). Further, inhibition of the proteasomal pathway with MG-132 blocked the degradation of BRD4, confirming that XZ1606 induces BRD4 degradation through the proteasome (Figure 2C). To investigate whether BRD4 is ubiquitinated before undergoing proteasomal degradation, we overexpressed BRD4 and ubiquitin (Figure 2D, left panel) and assessed the level of BRD4-associated ubiquitin in the presence of the proteasome inhibitor MG132. Treatment with XZ1606 led to an increase in overall ubiquitination levels (Figure 2D, right panel), suggesting that BRD4 is significantly ubiquitinated prior to proteasomal degradation. These results establish that XZ1606 degrades BRD4 in LX-2 cells via a ubiquitin-proteasomal pathway involving calcium signaling and ASGPR-mediated endocytosis. This mechanistic insight supports the potential of XZ1606 as a selective therapeutic agent for liver diseases, such as liver fibrosis, where BRD4 plays a crucial role. Overall, these findings show great anti-fibrotic potential for LIVTAC.

### LIVTAC inhibits fibrotic phenotypes in LX-2 cells induced by TGF-β via degrading BRD4

**Figure 3:**
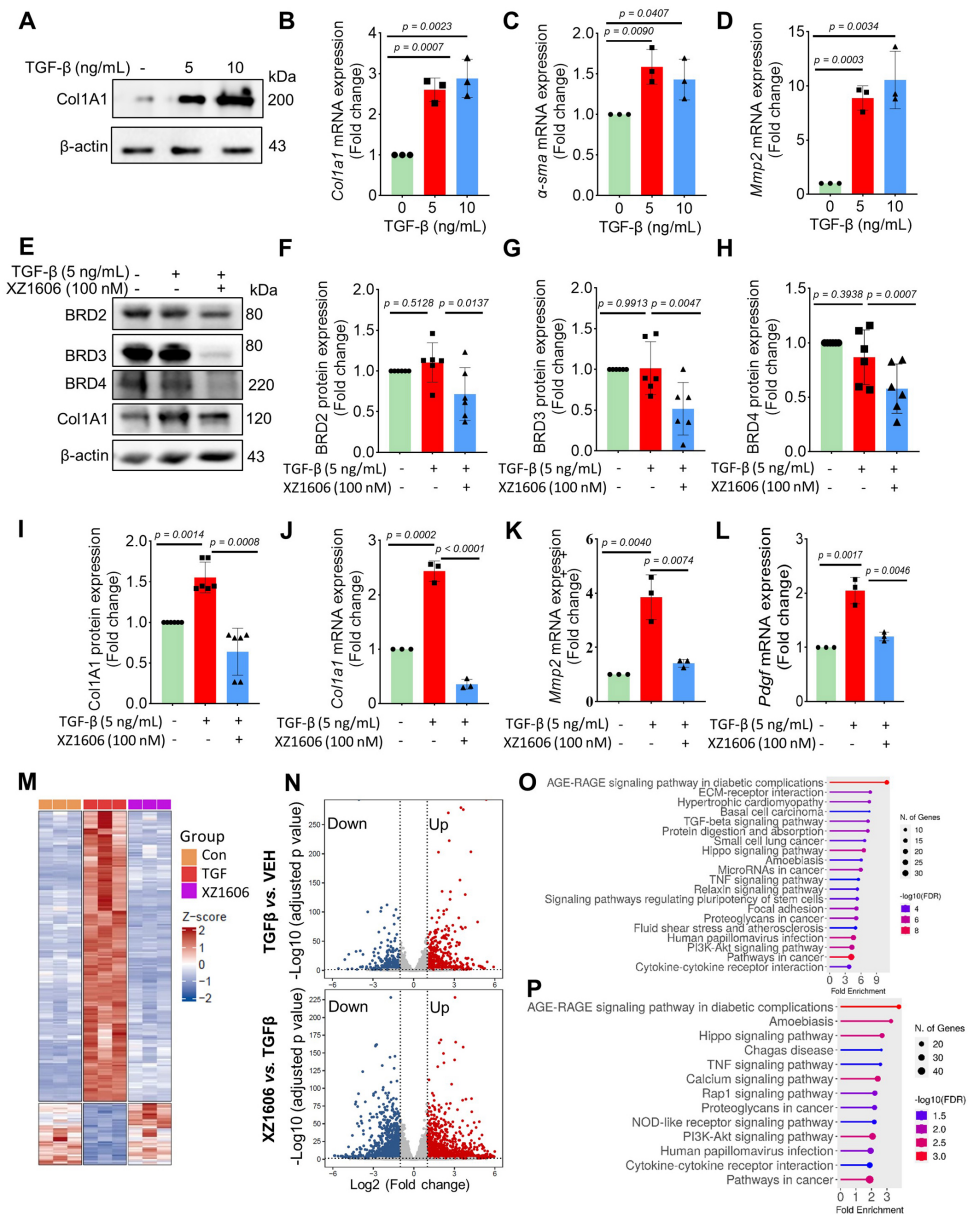
**Effect of XZ1606 on fibrosis markers in TGF-β-activated LX-2 cells. (A)** Western blot analysis showing the protein expression of the fibrotic marker Col1A1 in LX-2 cells treated with 5 ng/mL and 10 ng/mL of XZ1606. **(B-D)** mRNA expression levels of fibrotic markers *Col1a1*, *α-sma*, and *Mmp2* in LX-2 cells treated with 5 ng/mL and 10 ng/mL TGF-β, measured by qRT-PCR. Data are expressed as fold change relative to the control group, normalized to GAPDH expression (n = 3). **(E)** Western blot analysis showing the degradation of BRD2, BRD3, BRD4, and the downregulation of Col1A1 in LX-2 cells treated with 5 ng/mL TGF-β and 100 nM XZ1606. **(F-I)** Quantification of BRD2, BRD3, BRD4, and Col1A1 protein levels, normalized to β-actin, in control, TGF-β (5 ng/mL), and XZ1606 (100 nM) treatment groups (n = 6). **(J-L)** mRNA expression of *Col1a1, Mmp2, and Pdgf* in LX-2 cells treated with TGF-β (5 ng/mL) and XZ1606 (100 nM) (n = 3). **(M)** Hierarchical clustering of transcript expression data showing differential gene expression patterns across control, TGF-β, and XZ1606 treatment groups (n = 3). **(N)** Volcano plots showing differential gene expression between the TGF-β model vs. control and XZ1606 treatment vs. TGF-β model. Upregulated genes are shown in red and downregulated genes in blue. **(O)** KEGG pathway enrichment analysis of upregulated genes in the TGF-β model vs. control group, highlighting fibrotic pathways. **(P)** KEGG pathway enrichment analysis of downregulated genes in the XZ1606 treatment group vs. TGF-β model, showing the reversal of fibrotic pathways by XZ1606. Data are represented as the mean ± SEM, with statistical significance determined by one-way ANOVA followed by post-hoc comparisons (p < 0.05).

We evaluated the effect of XZ1606 on fibrotic markers in TGF-β-activated LX-2 cells to assess its potential anti-fibrotic effects. First, we confirmed that TGF-β treatment (5 and 10 ng/mL) upregulated the expression of Col1A1, a major fibrotic marker (Figure 3A). qRT-PCR analysis showed that TGF-β significantly increased the mRNA expression of fibrotic markers, including *Col1a1* (Figure 3B), *α-sma* (Figure 3C), and *Mmp2* (Figure 3D), compared to the control cells, suggesting successful construction of the fibrosis model *in vitro*. Western blot analysis revealed that XZ1606 treatment led to the degradation of BRD2, BRD3, and BRD4 and downregulated Col1A1 expression in TGF-β-stimulated LX-2 cells (Figure 3E-I). In addition to protein changes, mRNA levels of *Col1a1*, *Mmp2* and* Pdgf* were significantly reduced in the XZ1606 treatment group (Figure 3J-L), demonstrating the compound's ability to reverse TGF-β-induced fibrotic gene expression. These results suggest that XZ1606 targets multiple BET family proteins, including BRD4, and modulates key fibrotic pathways in LX-2 cells.

Transcriptomic analysis also revealed a distinct shift in gene expression patterns between the TGF-β model and the XZ1606 treatment group (Figure 3M-N). Hierarchical clustering of transcript expression data showed differential gene expression across the groups (Figure 3M). Further pairwise comparisons between the groups (File S1 & S2) identified upregulated and downregulated genes, which were visualized on volcano plots (Figure 3N). Notable upregulated genes in the TGF-β model compared to the control group included* Col1a2, Col4a1, Col3a1, Col4a2, Col5a3, Tgfb2, Tgfb1, Tgfbi, Vegfa, Mmp2, Mmp10, Smad7, Inhba, Gdf6, Bmpr1b, Wnt7a, Nox4, Ccl2, Fn1, Itgb6, Ngf, Ntf4, Pdgfb, Lpar5, Foxs1, Egr2, Foxp1-dt, C5ar2, Myct1, Edn1, Foxo1, Il6,* and* Pik3cd.* Functional enrichment analysis of upregulated genes in the TGF-β model (compared to the control group) revealed several fibrotic pathways, including AGE-RAGE signaling, TGF-β signaling, ECM-receptor interaction, Amoebiasis, Hippo signaling, PI3K-Akt signaling, and TNF signaling (Figure 3O). However, these pathways were downregulated in the XZ1606 treatment group (Figure 3P), providing further evidence that XZ1606 can reverse TGF-β-induced fibrotic changes. Overall, these findings demonstrate that XZ1606 inhibits fibrotic phenotypes in LX-2 cells induced by TGF-β by degrading BRD4 and other BET family proteins, thereby modulating key fibrotic signaling pathways and gene expression.

### LIVTAC inhibits hepatic fibrosis in mice induced by CCl₄

**Figure 4:**
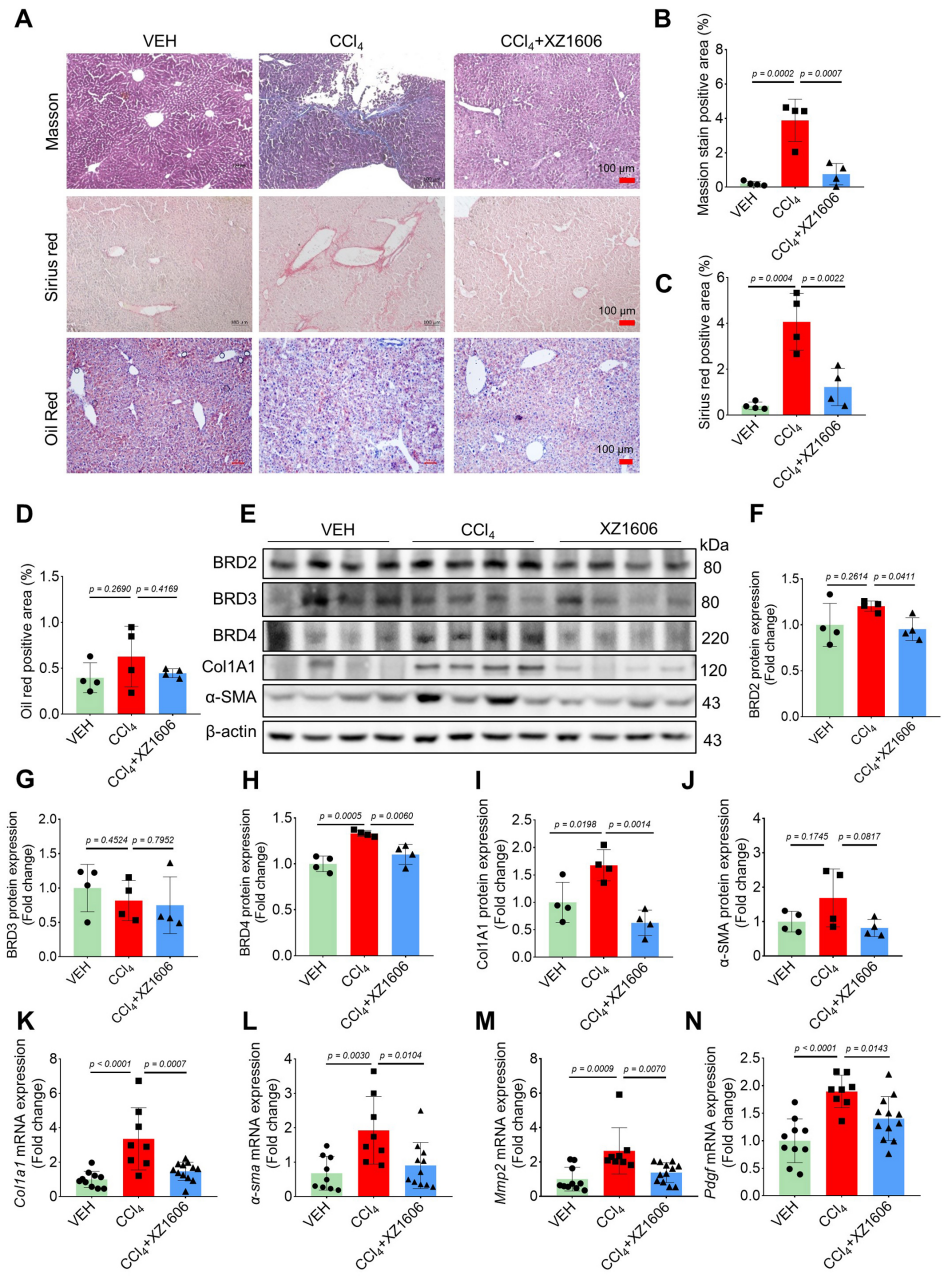
** Effect of XZ1606 treatment on liver fibrosis in the CCl₄-induced fibrosis model. (A)** Masson's trichrome, Sirius red, and Oil Red O staining of liver tissue sections were performed to assess collagen deposition and fibrosis severity. Representative images from the Vehicle (VEH), CCl₄, and CCl₄ + XZ1606 groups are shown. **(B)** Quantification of the positive staining area for Masson's trichrome (n = 4 for each group). **(C)** Quantification of Sirius red-positive areas (n = 4 for each group). **(D)** Quantification of Oil Red O-positive areas (n = 4 for each group). **(E)** Western blot analysis assessing the expression of BRD2, BRD3, BRD4, Col1A1, and α-SMA in liver tissue from the VEH, CCl₄, and CCl₄ + XZ1606 groups. The CCl₄ group displayed upregulation of fibrosis markers, including Col1A1 and α-SMA, whereas XZ1606 treatment resulted in a reduction in these markers, indicating decreased fibrosis. **(F-J)** Protein quantification of BRD2, BRD3, BRD4, α-SMA, and Col1A1 confirmed the downregulation of fibrotic markers in the CCl₄ + XZ1606 group compared to the CCl₄ group (n = 4 for each group). **(K-N)** qRT-PCR analysis of *Col1a1, α-sma, Mmp2,* and* Pdgf* mRNA expression levels in liver tissue from the VEH, CCl₄, and CCl₄ + XZ1606 groups. Data are presented as fold change relative to the VEH group, normalized to GAPDH expression, and represent the mean ± SEM (n = 10, 8 and 12, respectively). Statistical significance was determined by Student's t-test.

To assess the *in vivo* efficacy of XZ1606 in liver fibrosis, we established a CCl₄-induced mouse model of hepatic fibrosis. In a preliminary study, CCl₄-induced mice were treated with XZ1606 at doses of 0.5 and 1 mg/kg; however, these doses did not produce a significant therapeutic effect (Figure S2). Based on these findings, we selected 1.5 mg/kg as the optimal dose for subsequent experiments. The liver fibrosis model was induced by administering CCl₄ (1.25 mg/kg) for 6 weeks, and after the induction, the mice were treated with XZ1606 (1.5 mg/kg, intraperitoneally every 3 days) for 3 weeks (Figure S3A). CCl₄ is a widely used agent for inducing liver fibrosis and cirrhosis 43-45. Histological analysis of liver tissues from XZ1606-treated mice revealed a significant reduction in liver fibrosis compared to the CCl₄ group. This was evident from the Sirius red (red color), and Masson's trichrome staining (blue color) (Figure 4A-C), which are markers of collagen deposition and fibrosis 46-48. This liver fibrosis model results in liver injury and fibrosis; however, CCl₄ just slightly reduced body weight, without changes in other blood parameters (Figure S3B-I). There were no significant changes in lipid deposition or an imbalance in lipid metabolism, as evidenced by minimal Oil Red O staining in the liver (Figure 4A, 4D). The CCl₄-treated group exhibited strong positive staining indicative of extensive fibrosis, whereas the CCl₄ + XZ1606 treatment group showed significantly reduced staining, reflecting a decrease in collagen deposition and overall fibrosis severity (Figure 4A-C; Figure S3J). These results suggest that XZ1606 treatment mitigated fibrosis development in this model.

To further assess the therapeutic potential of XZ1606, protein expression levels of BET proteins and key fibrotic markers were analyzed. Among the BET proteins, only BRD4 was increased in the liver of mice treated with CCl₄, which was degraded by XZ1606 (Figure 4E-H), suggesting that BRD4 may be the major protein mediating fibrosis. Consistently, there was a marked reduction in the expression of Col1A1 and α-SMA, two important markers of fibrosis, in liver tissue from the XZ1606 treatment group (Figure 4E, I and J). These findings were supported by qRT-PCR analysis, which demonstrated significant downregulation of mRNA levels for *Col1a1, α-sma*, and other fibrosis-related genes, including *Mmp2* and *pdgf,* in the treated mice (Figure 4K-N). These results suggest that XZ1606 may exert its effects not only by reducing collagen deposition but also by modulating signaling pathways related to fibrosis progression.

**Figure 5:**
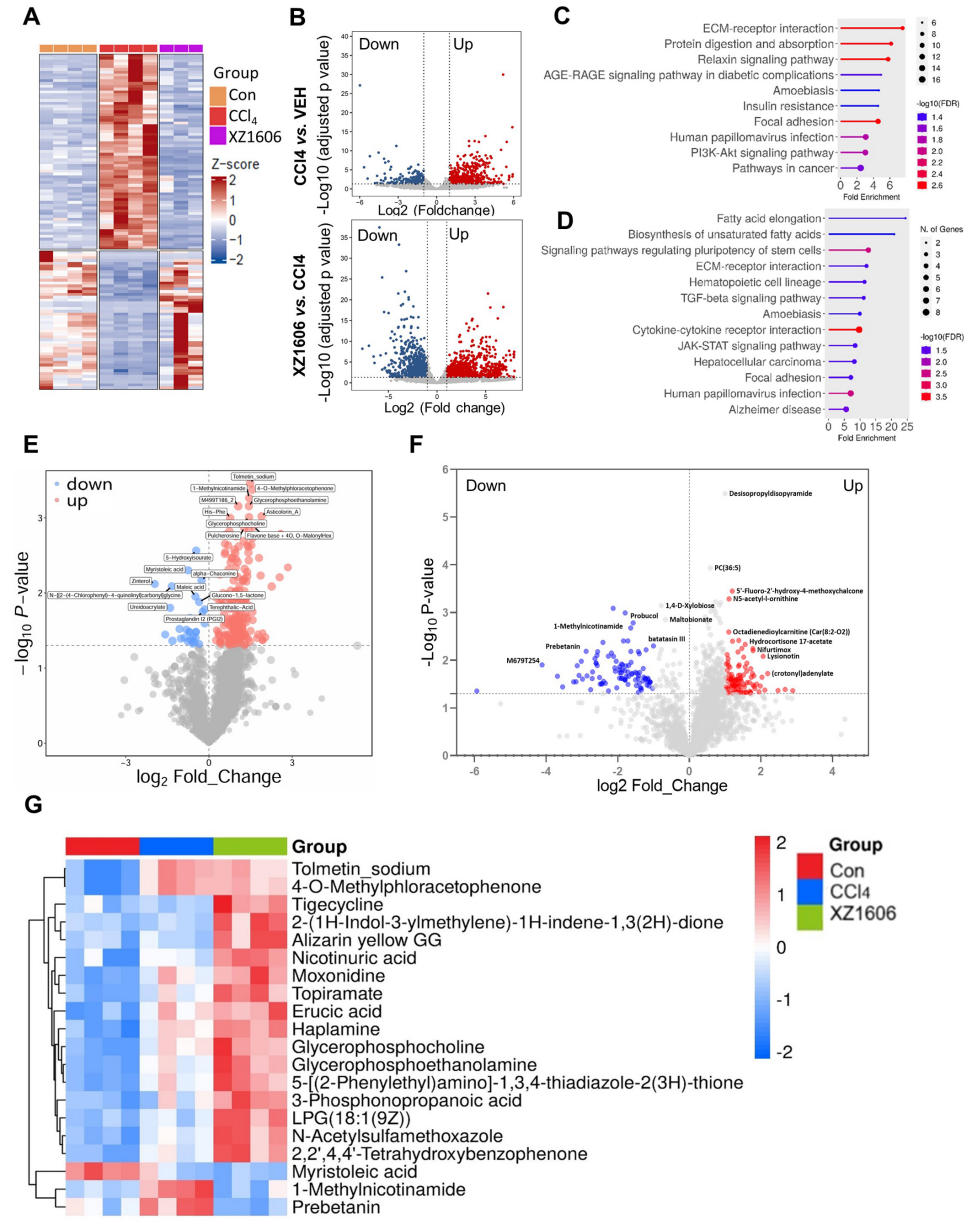
** Transcriptomic and metabolomic profiles under the XZ1606 treatment of liver fibrosis in the CCl₄-induced fibrosis model. (A-D)** Transcriptomic analysis (n = 4 for each group). **(A)** Hierarchical clustering of transcript expression data showing differential gene expression patterns across control, CCl_4_, and XZ1606 treatment groups. **(B)** Volcano plots showing differential gene expression between the CCl_4_ model vs. control and XZ1606 treatment vs. CCl_4_ model. Upregulated genes are shown in red and downregulated genes in blue.** (C)** KEGG pathway enrichment analysis of upregulated genes in the CCl_4_ model vs. control group, highlighting fibrotic pathways. **(D)** KEGG pathway enrichment analysis of downregulated genes in the XZ1606 treatment group vs. CCl_4_ model, showing the reversal of fibrotic pathways by XZ1606. **(E-G)** Metabolomic analysis (n = 4 for each group). **(E)** Volcano plots showing differential metabolite expression between the CCl_4_ model vs. control. **(F)** Volcano plots showing differential metabolite expression between the XZ1606 treatment vs. CCl_4_ model. **(G)** Hierarchical clustering of metabolomics data showing differential metabolite expression patterns across the groups.

To evaluate the antifibrotic effects of XZ1606 in a CCl₄-induced liver fibrosis model, we conducted both transcriptomic and metabolomic analyses (Figure 5; File S3-4). Hierarchical clustering of RNA-sequencing data revealed distinct gene expression patterns among the control, CCl₄, and XZ1606 treatment groups (Figure 5A). Notably, the CCl₄-treated mice formed a cluster separate from the controls, indicating significant transcriptional shifts under fibrotic conditions. However, mice receiving XZ1606 treatment displayed a partial reversion toward the control-like expression profile. Volcano plots further highlighted widespread gene upregulation (red) and downregulation (blue) in CCl₄-treated mice compared to controls (Figure 5B). Upon XZ1606 administration, many of these differentially expressed genes shifted back, indicating a reversal of the fibrotic gene signature. KEGG pathway enrichment analysis showed that upregulated genes in the CCl₄ model primarily mapped to fibrotic and inflammatory pathways (Figure 5C), including ECM-receptor interaction and AGE-RAGE signaling 49, 50. By contrast, in the XZ1606 treatment group (Figure 5D), these same pathways were among the most downregulated, suggesting that LIVTAC counters the profibrotic transcriptional program induced by CCl₄.

Volcano plots of metabolite changes revealed multiple upregulated (red) and downregulated (blue) metabolites in the CCl₄ model compared to control mice (Figure 5E), consistent with the metabolic dysregulation associated with fibrosis. Following XZ1606 administration, many of these metabolite alterations were reversed (Figure 5F), indicating that XZ1606 not only modulates fibrotic gene expression but also restores metabolic homeostasis. Hierarchical clustering of metabolomic profiles also suggests that some metabolite patterns, such as 1-Methylnicotinamide and Prebetanin (anti-inflammatory and antioxidant) (Figure 5G), were shifted closer to the control group. Additionally, Topiramate, which was upregulated in the treatment group, may serve as an adjunct to lifestyle interventions in the treatment of pediatric metabolic dysfunction associated steatotic liver disease (MASLD), particularly in cases where lifestyle modifications are insufficient or other obesity pharmacotherapies are not tolerated or applicable. Collectively, these transcriptomic and metabolomic findings demonstrate that XZ1606 effectively alleviates the molecular and metabolic hallmarks of CCl₄-induced hepatic fibrosis.

### LIVTAC inhibits hepatic steatosis and fibrosis in mice induced by choline-deficient L-amino acid defined-high fat diet (CDAA-HFD)

**Figure 6:**
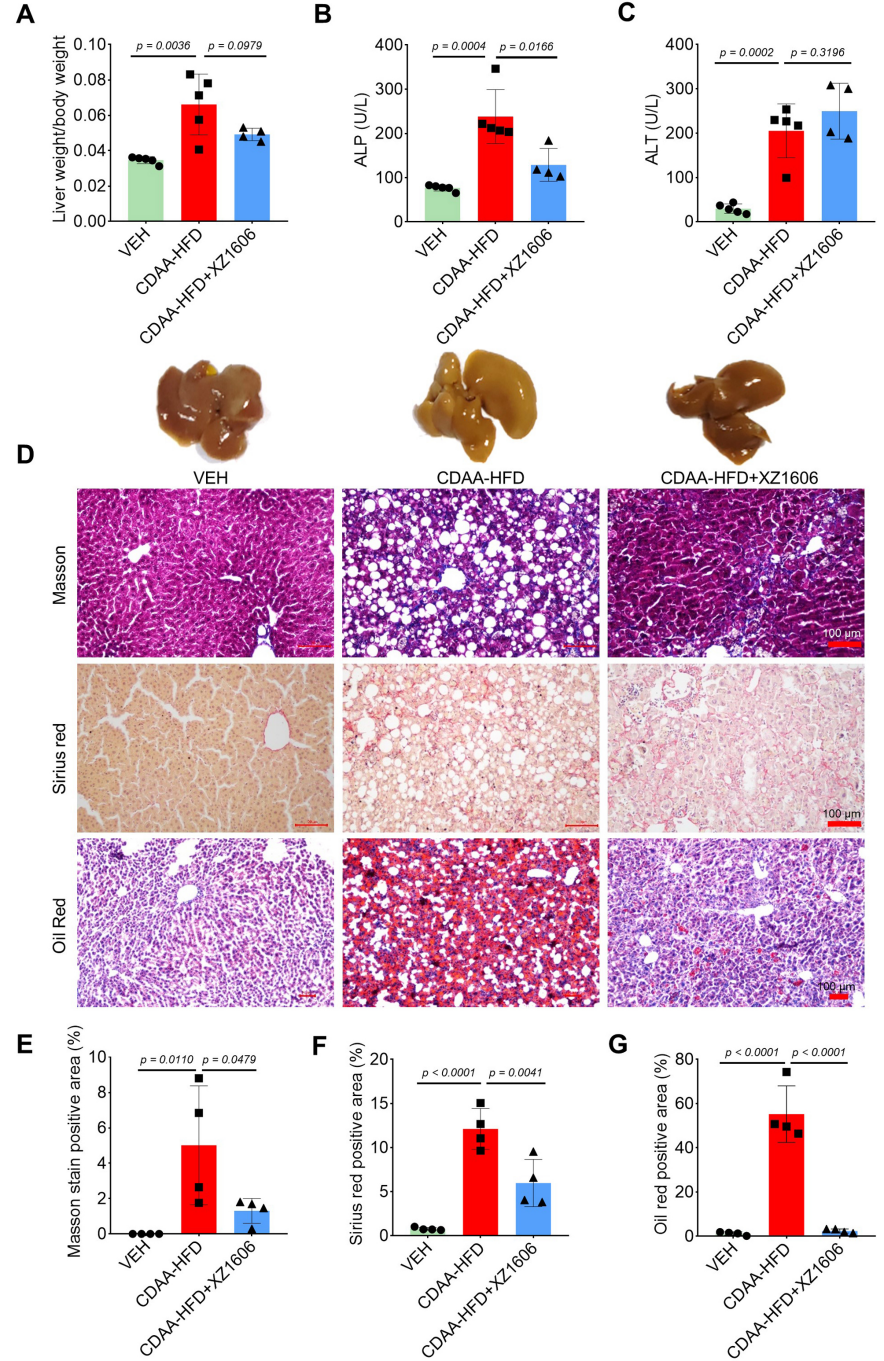
** Effect of XZ1606 treatment on hepatic steatosis and fibrosis in the CDAA-HFD model. (A)** The ratio of liver weight to body weight in vehicle (VEH), CDAA-HFD, and CDAA-HFD + XZ1606 groups. **(B)** Serum alkaline phosphatase (ALP) levels across the three groups, indicating liver injury. **(C)** Serum alanine aminotransferase (ALT) levels, another marker of liver damage. **(D)** Representative images of liver sections stained with Masson's trichrome, Sirius red, and Oil Red O to evaluate fibrosis, collagen deposition, and lipid accumulation, respectively. **(E-G)** Quantification of the positively stained areas for Masson's trichrome **(E)**, Sirius red **(F)**, and Oil Red O **(G)**. Mice on the CDAA-HFD show significant increases in fibrosis and steatosis, whereas XZ1606 treatment markedly reduces these pathological features. Data are presented as mean ± SEM (n = 5, 5 and 4 mice for VEH, CDAA-HFD and CDAA-HFD + XZ1606 groups, respectively), with statistical significance determined by one-way ANOVA followed by post-hoc comparisons (p < 0.05).

Building on our findings in the CCl₄-induced fibrosis model, where we identified 1.5 mg/kg as an effective dose of XZ1606, we next assessed whether this same dose could mitigate liver steatosis and fibrosis in a CDAA-HFD mouse model (Figure S4). In this model, mice were fed a choline-deficient, L-amino acid-defined, high-fat diet for 10 weeks to induce liver injury 51 characterized by both steatosis and fibrosis. Subsequently, animals received intraperitoneal injections of XZ1606 (1.5 mg/kg) every 3 days for 4 weeks. After establishing the CDAA-HFD mouse model (Figure S4A), we then further assessed the impact of XZ1606 on hepatic injury and fibrosis in this model. As shown in Figure 6A and Figure S4B-E, the CDAA-HFD group displayed a significant increase in the liver weight to body weight ratio compared with vehicle controls, consistent with hepatomegaly and fat accumulation. Treatment with XZ1606 partially reversed this effect, suggesting an improvement in overall liver health. Serum ALP and ALT levels were also elevated in the CDAA-HFD group (Figure 6B, C), indicative of liver injury 52. Although ALT remained somewhat higher in XZ1606-treated mice, ALP was significantly reduced, reflecting a decrease in liver damage. Histological staining further corroborated these biochemical findings: the CDAA-HFD group exhibited pronounced collagen deposition and lipid accumulation, as evidenced by intense Masson's trichrome, Sirius red, and Oil Red O staining (Figure 6D). In contrast, XZ1606-treated mice showed markedly reduced staining intensities, implying less fibrosis and steatosis. Quantitative analysis of positively stained areas demonstrated that XZ1606 treatment lowered both the fibrotic burden (Masson's trichrome, Sirius red) and lipid accumulation (Oil Red O) compared to the CDAA-HFD group (Figure 6E-G).

**Figure 7:**
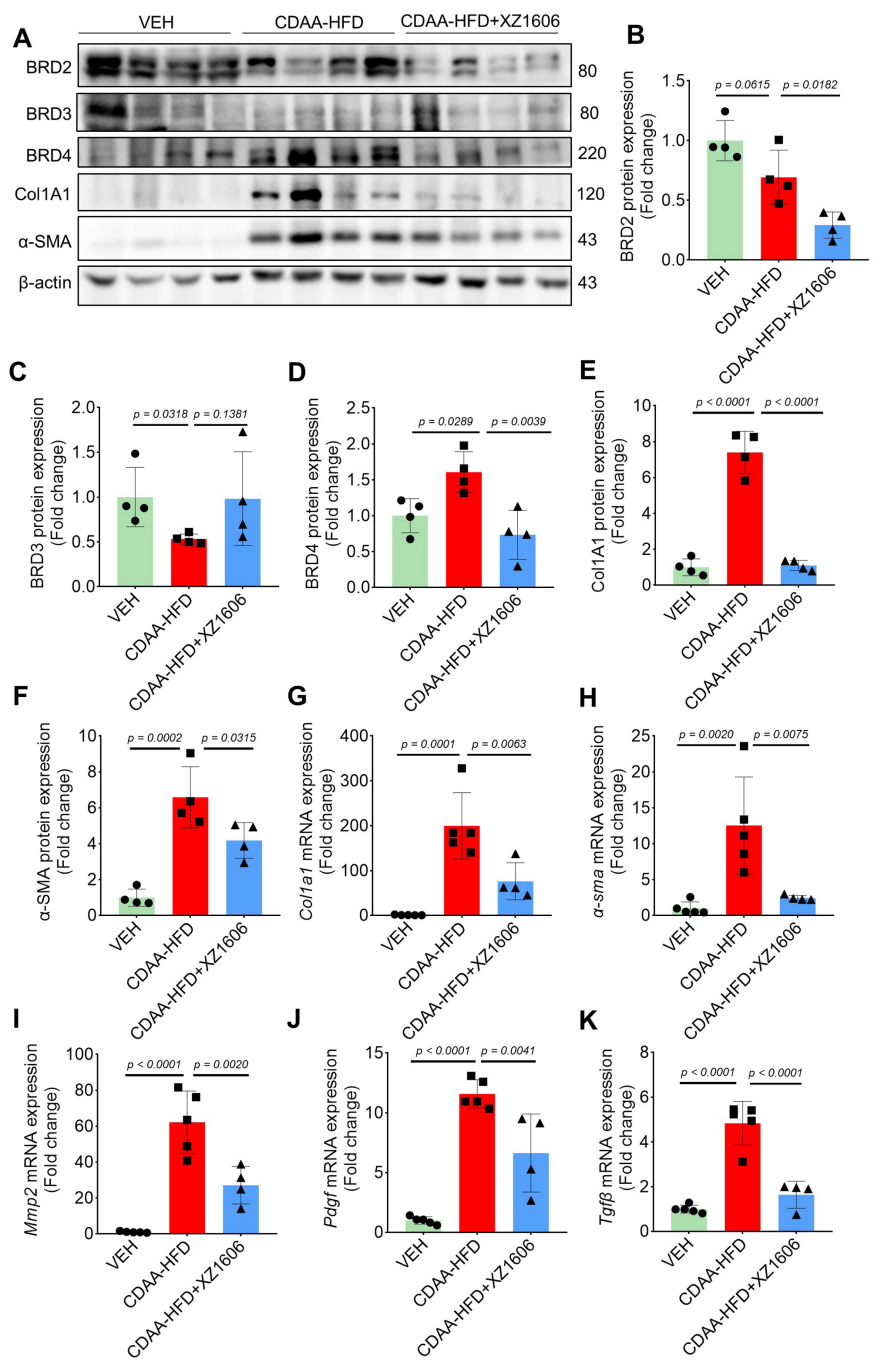
***In vivo* anti-fibrotic effects of XZ1606 in the HFD-CDAA liver fibrosis mouse model. (A)** Western blot analysis of BRD2, BRD3, BRD4, α-SMA, and Col1A1 protein expression in liver tissue from Vehicle (VEH), CDAA-HFD, and CDAA-HFD + XZ1606-treated mice. Representative blots show that fibrosis-related proteins were upregulated in the CDAA-HFD group and significantly downregulated in the CDAA-HFD + XZ1606 treatment group. **(B-F)** Quantification of BRD2, BRD3, BRD4, Col1A1, and α-SMA protein expressions in the CDAA-HFD + XZ1606 treatment groups (n = 4). **(G-K)** qRT-PCR analysis of mRNA expression levels of fibrotic markers: *Col1a1, α-sma, Mmp2, Pdgf, and Tgfβ* in liver tissue from the three experimental groups. Data are presented as mean ± SEM (n = 5, 5 and 4, respectively), with statistical significance determined by one-way ANOVA followed by post-hoc comparisons.

Furthermore, Western blot analysis (Figure 7A) of liver tissues revealed elevated expression of BRD4, but not BRD2 and BRD3 in the CDAA-HFD group (Figure 7A-D), again suggesting BRD4 as the major protein that mediates liver fibrosis. The fibrosis-related proteins, including Col1A1, and α-SMA in the CDAA-HFD group were all downregulated by the treatment of XZ1606 (Figure 7B-G), further supporting its anti-fibrotic effects. To complement these findings, qRT-PCR analysis (Figure 7H-K) of liver tissues showed that the CDAA-HFD group had elevated mRNA levels for key fibrosis markers such as *Col1a1*, *α-sma*, *Tgfb*, and* Mmp2*, indicative of significant fibrosis progression. Treatment with XZ1606 resulted in a marked reduction of these mRNA levels (Figure 7H-K). These molecular findings suggest that XZ1606 treatment effectively modulates the fibrotic pathways and reduces the expression of genes involved in fibrosis and tissue remodeling.

**Figure 8:**
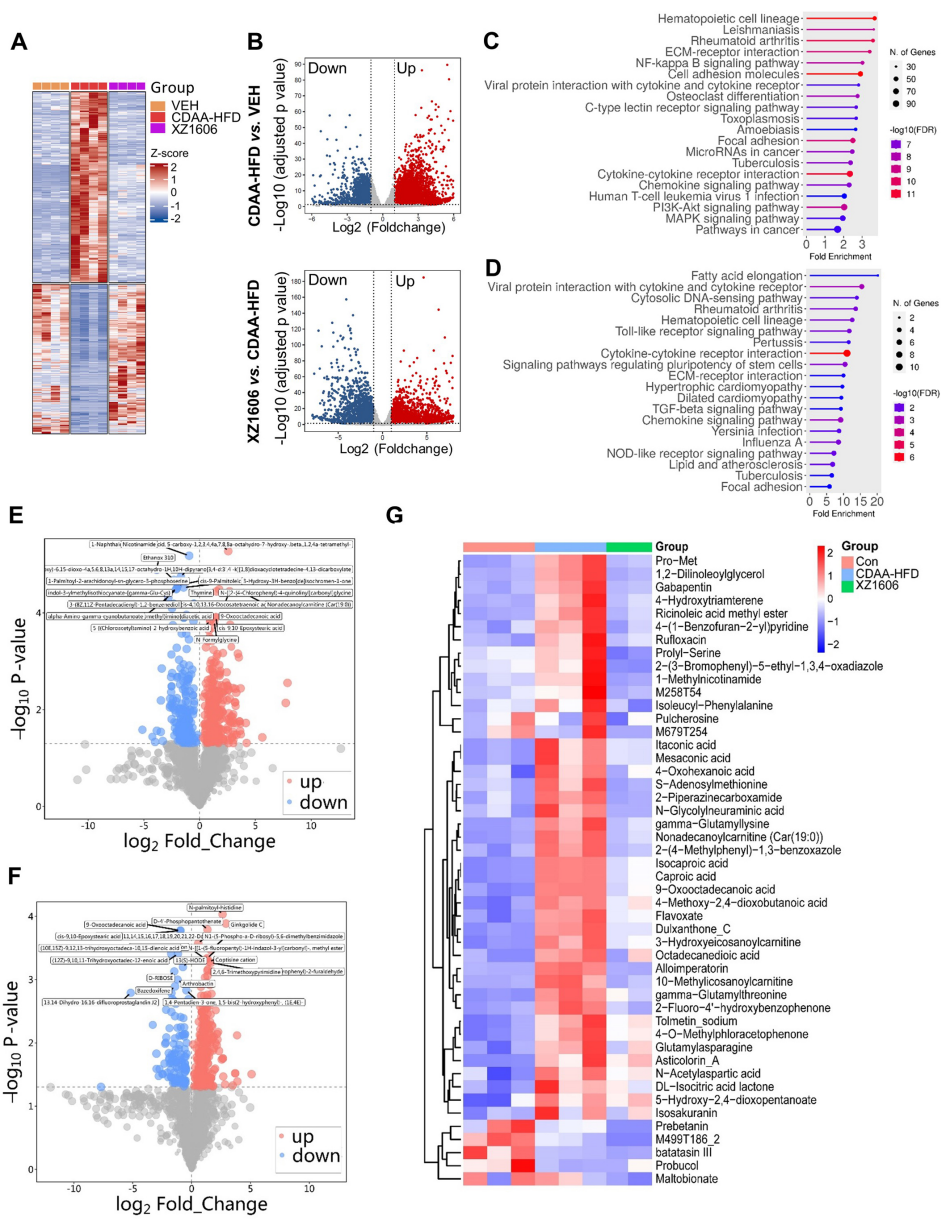
** Transcriptomic and metabolomic profiles under the XZ1606 treatment of liver fibrosis in the CDAA-HFD fibrosis model. (A-D)** Transcriptomic analysis. **(A)** Hierarchical clustering of transcript expression data showing differential gene expression patterns across control, CDAA-HFD, and XZ1606 treatment groups (n = 4 for each group). **(B)** Volcano plots showing differential gene expression between the CDAA-HFD model vs. control and XZ1606 treatment vs. CDAA-HFD model. Upregulated genes are shown in red and downregulated genes in blue. **(C)** KEGG pathway enrichment analysis of upregulated genes in the CDAA-HFD model vs. control group, highlighting fibrotic pathways. **(D)** KEGG pathway enrichment analysis of downregulated genes in the XZ1606 treatment group vs. CDAA-HFD model, showing the reversal of fibrotic pathways by XZ1606. **(E-G)** Metabolomic analysis (n = 3, 3 and 2 samples for control, CDAA-HFD and XZ1606 treatment groups, respectively). One sample from the treatment group (originally n = 3) did not pass the quality control and was removed from analysis. **(E)** Volcano plots showing differential metabolite expression between the CDAA-HFD model vs. control. **(F)** Volcano plots showing differential metabolite expression between the XZ1606 treatment vs. CDAA-HFD model. **(G)** Hierarchical clustering of metabolomics data showing differential metabolite expression patterns across the groups.

To gain deeper insights into the molecular and metabolic changes underlying XZ1606's protective effects in the CDAA-HFD model, we performed comprehensive transcriptomic and metabolomic analyses (Figure 8; File S5-6). Hierarchical clustering of RNA-sequencing data revealed a distinct gene expression pattern in the CDAA-HFD group compared to controls (Figure 8A), characterized by the upregulation of numerous fibrotic and inflammatory genes. The XZ1606-treated group displayed a partial shift toward the control-like profile, suggesting that LIVTAC attenuates or reverses the pathological gene signature induced by the diet. Volcano plots further confirm these trends: the top panel (CDAA-HFD vs. control) shows substantial upregulation (red) and downregulation (blue) of genes in the fibrotic state (Figure 8B), whereas the bottom panel (XZ1606 vs. CDAA-HFD) indicates that many of these dysregulated genes return to near-control levels following treatment. Consistent with this observation, KEGG pathway enrichment revealed that genes elevated in the CDAA-HFD group are associated with profibrotic and inflammatory signaling (Figure 8C). In contrast, these same pathways are among the most downregulated in XZ1606-treated mice (Figure 8D), supporting the idea that LIVTAC counteracts the transcriptional drivers of fibrosis. Analysis of the downregulated genes in the XZ1606 treatment group reveals a broad attenuation of proinflammatory and profibrotic signaling pathways. Notably, several chemokines and cytokines (e.g., *Cxcl1*, *Cxcl11*, *Ccl2*, *Ccl5*, *Il1a*, *Il1r1*) and interferon-induced genes (*Ifi27*, *Ifi35*, *Irf3*, *Irf7*) were suppressed, suggesting a reduction in immune cell recruitment and inflammatory responses. Key fibrotic mediators—such as collagens (*Col5a3*, *Col24a1*, *Col18a1*), TGF-β family members (*Inhba*, *Inhbc*, *Inhbe*), and NADPH oxidase 4 (*Nox4*) also show decreased expression, aligning with an inhibition of extracellular matrix deposition and oxidative stress. Downregulation of *Wnt5b* indicates diminished pro-fibrotic Wnt signaling, while reduced levels of matrix metalloproteinases (*Mmp12*, *Mmp15*, *Mmp27*) and integrins (*Itga4*, *Itga5*, *Itgax*) suggest that both ECM remodeling and cell adhesion processes are tempered under treatment. Moreover, genes linked to metabolic dysregulation (*Elovl3, Elovl5*, involved in fatty acid elongation) and immune-related C-type lectins (*Clec2d*, *Clec2h*, *Clec4b1*, *Clec7a*, *Clec9a*) are also suppressed, indicating a broad-spectrum normalization of pathologic pathways. Collectively, this pattern of downregulated genes underscores XZ1606's capacity to curb multiple facets of the fibrotic and inflammatory cascade in liver disease.

Metabolomic profiling reinforced the transcriptomic findings. Volcano plots (Figure 8E-F; File S7) illustrate widespread metabolic disturbances in the CDAA-HFD group, with numerous metabolites showing significant increases or decreases compared to controls. Upon XZ1606 treatment, many of these altered metabolites shifted back toward control levels, indicative of restored metabolic balance. Hierarchical clustering of the metabolite data confirms that the XZ1606-treated group more closely resembles the control group's profile, whereas the CDAA-HFD group remains distinctly separated (Figure 8G). Analysis of the metabolites downregulated in the XZ1606 treatment group indicates a broad normalization of hepatic metabolic processes that are otherwise perturbed during fibrosis. Many of the listed molecules, such as 1,2-Dilinoleoylglycerol, Nonadecanoylcarnitine, and 3-Hydroxyeicosanoylcarnitine, are involved in lipid metabolism, suggesting that XZ1606 may reduce excessive lipid accumulation or abnormal fatty acid oxidation associated with fibrotic conditions. The decrease in S-Adenosylmethionine (a central methyl donor) and Itaconic acid (an immunometabolite) further points to dampened inflammatory or stress-related pathways in the liver. Of particular note, 1-Methylnicotinamide, a metabolite of nicotinamide that was elevated in both the CCl₄- and CDAA-HFD induced fibrosis models, consistently declined in the treatment group. This downregulation underscores XZ1606's potential to restore NAD⁺-related metabolic balance and reduce the proinflammatory environment often linked to high 1-Methylnicotinamide levels. Taken together, these shifts in the metabolome reinforce the conclusion that XZ1606 not only modulates fibrotic gene expression but also helps reestablish metabolic homeostasis in fibrotic livers.

Overall, these transcriptomic and metabolomic results align with the histological and biochemical evidence of reduced fibrosis and steatosis. By reversing both gene expression and metabolic abnormalities, XZ1606 demonstrates a broad-spectrum capacity to mitigate the fibrotic and steatotic changes characteristic of the CDAA-HFD model.

## Discussion

Liver fibrosis is a complex and dynamic process characterized by the excessive accumulation of extracellular matrix components, which can ultimately progress to cirrhosis and HCC 53-55. Despite significant advances in understanding the molecular mechanisms driving fibrogenesis, effective therapies that selectively target the underlying pathology without causing off-target toxicity remain elusive. In this study, we repurposed the LIVTAC XZ1606, originally developed for HCC, to treat liver fibrosis by inducing selective degradation of BRD4, a bromodomain-containing protein known to regulate fibrotic gene expression 25, 56. Our multifaceted approach, incorporating *in vitro* cellular assays, *in vivo* animal models, and comprehensive omics analyses, demonstrates that LIVTAC XZ1606 effectively reverses fibrotic and steatotic changes in the liver through a highly selective and potent mechanism.

Our *in vitro* experiments in LX-2 cells, a well-established human HSC line 57, 58 provided critical proof-of-concept for the antifibrotic efficacy of XZ1606. We observed that XZ1606 induced robust and dose-dependent degradation of BRD4 at sub-nanomolar concentrations, with an IC₅₀ of less than 10 nM achieved within 24 h. The time-course analysis revealed that maximal BRD4 degradation occurred within 12 h of treatment, and this effect was sustained over an extended period. Importantly, our mechanistic studies confirmed that the degradation process was mediated by the ubiquitin-proteasomal pathway 59, as evidenced by the complete inhibition of BRD4 degradation upon treatment with the proteasomal inhibitor MG-132.

Furthermore, the partial inhibition of BRD4 degradation by the calcium chelator EGTA underscores the role of calcium signaling in the endocytic uptake of XZ1606. Specifically, the degradation of BRD4 by XZ1606 was blocked by shRNA-mediated ASGPR knockdown. Together, these findings validate the proposed mechanism of action and establish the high potency and specificity of XZ1606 in targeting fibrotic pathways in HSCs. At the core of our strategy is the unique design of XZ1606, which couples a potent GNE-987-derived BET degrader to a tri-GalNAc moiety. This design leverages the high expression of the ASGPR on HSCs to ensure liver-specific uptake via receptor-mediated endocytosis. Once internalized, the Val-Ala-PAB linker is cleaved by cathepsin B, a lysosomal enzyme, releasing the active PROTAC that recruits an E3 ligase to target BRD4 for ubiquitination and proteasomal degradation. This targeted degradation strategy not only results in the selective depletion of BRD4 in HSCs but also circumvents the “on-target off-tissue” toxicities commonly associated with systemic BET inhibitors 60-63.

In addition to *in vitro* data, our *in vivo* studies in two distinct mouse models of liver fibrosis further underscore the therapeutic potential of XZ1606. In the widely used CCl₄-induced liver fibrosis model 43, administration of CCl₄ over six weeks resulted in pronounced fibrosis, as evidenced by increased collagen deposition, elevated liver injury markers, and significant transcriptional alterations 64, 65. Treatment with XZ1606 (1.5 mg/kg, administered intraperitoneally every three days) for three weeks after fibrosis induction led to marked improvements in liver histology. Sirius red and Masson's trichrome staining revealed a significant reduction in collagen deposition in the treatment group compared to the CCl₄-only group. Moreover, immunoblotting and qRT-PCR analyses demonstrated substantial downregulation of key fibrotic markers such as *Col1a1, α-sma,* and* Tgfb,* alongside reductions in the expression of BRD4. These results indicate that XZ1606 not only suppresses the fibrotic gene signature but also directly modulates the molecular pathways that drive fibrosis.

Building on the promising results from the CCl₄ model, we extended our investigation to a choline-deficient, L-amino acid-defined high-fat diet (CDAA-HFD) model, which more closely mimics the clinical scenario of nonalcoholic steatohepatitis (NASH)-related fibrosis 66, 67. In the CDAA-HFD model, mice developed significant hepatomegaly, steatosis, and fibrosis over a 10-week feeding period. Subsequent treatment with XZ1606 for four weeks resulted in a partial reversal of these pathological features. Specifically, XZ1606-treated mice exhibited reduced liver weight-to-body weight ratios and improved serum biochemical parameters, such as lower ALP levels. Histologically, there was a significant reduction in both collagen deposition and lipid accumulation, as evidenced by diminished staining intensity in Masson's trichrome, Sirius red, and Oil Red O assays. These improvements were further supported by molecular analyses, which showed significant downregulation of fibrosis-related proteins and genes in the liver tissue of XZ1606-treated mice.

To gain deeper insight into the molecular and metabolic underpinnings of XZ1606's antifibrotic effects, we performed comprehensive transcriptomic and metabolomic analyses in both the CCl₄ and CDAA-HFD models. Transcriptomic profiling revealed that XZ1606 treatment partially reverted the fibrotic gene expression signature toward a control-like state. Notably, KEGG pathway enrichment analysis demonstrated that pro-fibrotic pathways, such as ECM-receptor interaction, AGE-RAGE signaling, and TGF-β signaling 68-73, were significantly downregulated following XZ1606 treatment. Similarly, metabolomic analysis showed that many metabolites associated with dysregulated lipid metabolism and inflammatory responses were restored to near-normal levels in the XZ1606-treated groups. Of particular interest was the consistent downregulation of 1-Methylnicotinamide 74-76, underscoring the compound's role in restoring NAD⁺-related metabolic balance.

Our study thus provides a comprehensive demonstration that targeted degradation of BRD4 using a LIVTAC is an effective strategy to reverse both fibrotic and steatotic changes in the liver. The dual approach of transcriptomic and metabolomic profiling not only corroborates the histological and biochemical improvements observed in our animal models but also offers novel insights into the underlying mechanisms by which XZ1606 exerts its therapeutic effects.

Despite these promising findings, several limitations warrant discussion. First, while our preclinical models provide robust evidence of XZ1606's efficacy, further studies are required to assess its long-term safety and potential immunogenicity in larger animal models. Additionally, the optimal dosing regimen and potential combination strategies with other antifibrotic agents remain to be explored. Furthermore, while XZ1606 effectively induces BRD4 degradation, its potential cytotoxic effects on non-target cells and overall impact on cellular viability require further investigation. Finally, although we focused primarily on BRD4, the contribution of other BET family members and potential off-target effects needs further clarification to fully understand the therapeutic window of LIVTAC XZ1606. Furthermore, the long-term safety and efficacy of LIVTACs in chronic liver disease models remain underexplored. A comprehensive evaluation of these limitations is essential for optimizing their design and translating preclinical success into clinical application.

In conclusion, this study introduces LIVTAC XZ1606 as a novel and selective therapeutic agent capable of reversing hepatic fibrosis and steatosis through targeted degradation of BRD4. These findings open new avenues for the treatment of chronic liver diseases, offering the potential for improved clinical outcomes with minimized systemic toxicity. Future studies will be crucial to translate these preclinical insights into viable clinical therapies for patients suffering from liver fibrosis and related disorders.

## Supplementary Material

Supplementary figures and tables.

## Abbreviations

ALP: alkaline phosphatase
ALT: alanine transaminase
ANOVA: analysis of variance
ASGPR: asialoglycoprotein receptor
ATCC: American Type Culture Collection
ATII: alveolar type II
BCA: bicinchoninic acid
CCl_4_: carbon tetrachloride
CDAA-HFD: choline-deficient L-amino acid-defined high-fat diet
DC_50_: half-maximal degradation concentration
DEGs: differentially expressed genes
DMEM: Dulbecco's Modified Eagle Medium
ECM: extracellular matrix
ESI: electrospray ionization
FBS: fetal bovine serum
GalNAc: *N*-acetylgalactosamine
HCC: hepatocellular carcinoma
HDF: human dermal fibroblasts
HELF: human lung embryo fibroblast
HSC: hepatic stellate cell
IDA: information-dependent acquisition
LC-MS: liquid chromatography-tandem mass spectrometry
LIVTAC: liver-targeting chimera
NASH: nonalcoholic steatohepatitis
NGDC: National Genomics Data Center
OPLS-DA: orthogonal projections to latent structures-discriminant analysis
PBS: phosphate-buffered saline
PCA: principal component analysis
PVDF: polyvinylidene fluoride
QC: quality control
qRT-PCR: quantitative Real-Time polymerase chain reaction
SEM: standard error of the mean
SPF: specific pathogen-free
TBS-T: tris-buffered saline with 0.1% Tween-20
TGF-β: transforming growth factor beta
THPA: The Human Protein Atlas
TPM: transcript per million
VIP: variable importance in the projection
