# Supplementary material for: Liver-targeted degradation of BRD4 reverses hepatic fibrosis and enhances metabolism in murine models
Source: Theranostics. 2025 Jun 18;15(15):7270–90. doi: 10.7150/thno.113852 (PMC12315693; doi:10.7150/thno.113852)
Supplement: Supplementary file 1 — Supplementary figures and tables. [file thnov15p7270s1.zip › 3-Supplementary Tables.pdf]

Table S1. Primary antibodies for Western blotting.

Antibody	Cat No.	Company
COL1A1	HY-P81227	MCE
α-SMA	14395-1-AP	Proteintech
BRD2	144-64229-100	RayBiotech
BRD3	sc-81202	SANTA
BRD4	PA5-100998	Invitrogen
BRD4	PA5-85662	Invitrogen
β-actin	AF5003	Beyotime
ASGPR	66692-1-Ig	Proteintech

Table S2. Primer pairs for qRT-PCR.

Gene name (species)		Sequence 5'-3'
α-sma (human)	Forward	CCTTGTTTGGGAAGCAAGTGG
	Reverse	TGGAGCTGCTTCACAGGATT
α-sma (mice)	Forward	CCCAGACATCAGGGAGTAATGG
	Reverse	TCTATCGGATACTTCAGCGTCA
Col1a1 (human)	Forward	GTGCGATGACGTGATCTGTGA
	Reverse	CGGTGGTTTCTTGGTCGGT
Col1a1 (mice)	Forward	TAAGGGTCCCCAATGGTGAGA
	Reverse	GGGTCCCTCGACTCCTACAT
Timp1 (mice)	Forward	TGGGAAATGCCGCAGATA
	Reverse	GCCAGGGAACCAAGAAGC
Mmp2 (human)	Forward	TACAGGATCATTGGCTACACACC
	Reverse	GGTCACATCGCTCCAGACT
Mmp2 (mice)	Forward	CCCCGATGCTGATACTGA
	Reverse	CTGTCCGCCAAATAAACC
Pdgf (human)	Forward	CAGCGACTCCTGGAGATAGAC
	Reverse	GGACAGCTTCCTCGATGCTT
Pdgf (mice)	Forward	GCGGGCTACTATACTATGCG
	Reverse	GATGACGGTAAGGACCACTAA
