# Supplementary material for: Liver-targeted degradation of BRD4 reverses hepatic fibrosis and enhances metabolism in murine models
Source: Theranostics. 2025 Jun 18;15(15):7270–90. doi: 10.7150/thno.113852 (PMC12315693; doi:10.7150/thno.113852)
Supplement: Supplementary file 1 — Supplementary figures and tables. [file thnov15p7270s1.zip › 2--Supplementary Figures-Final.pdf]

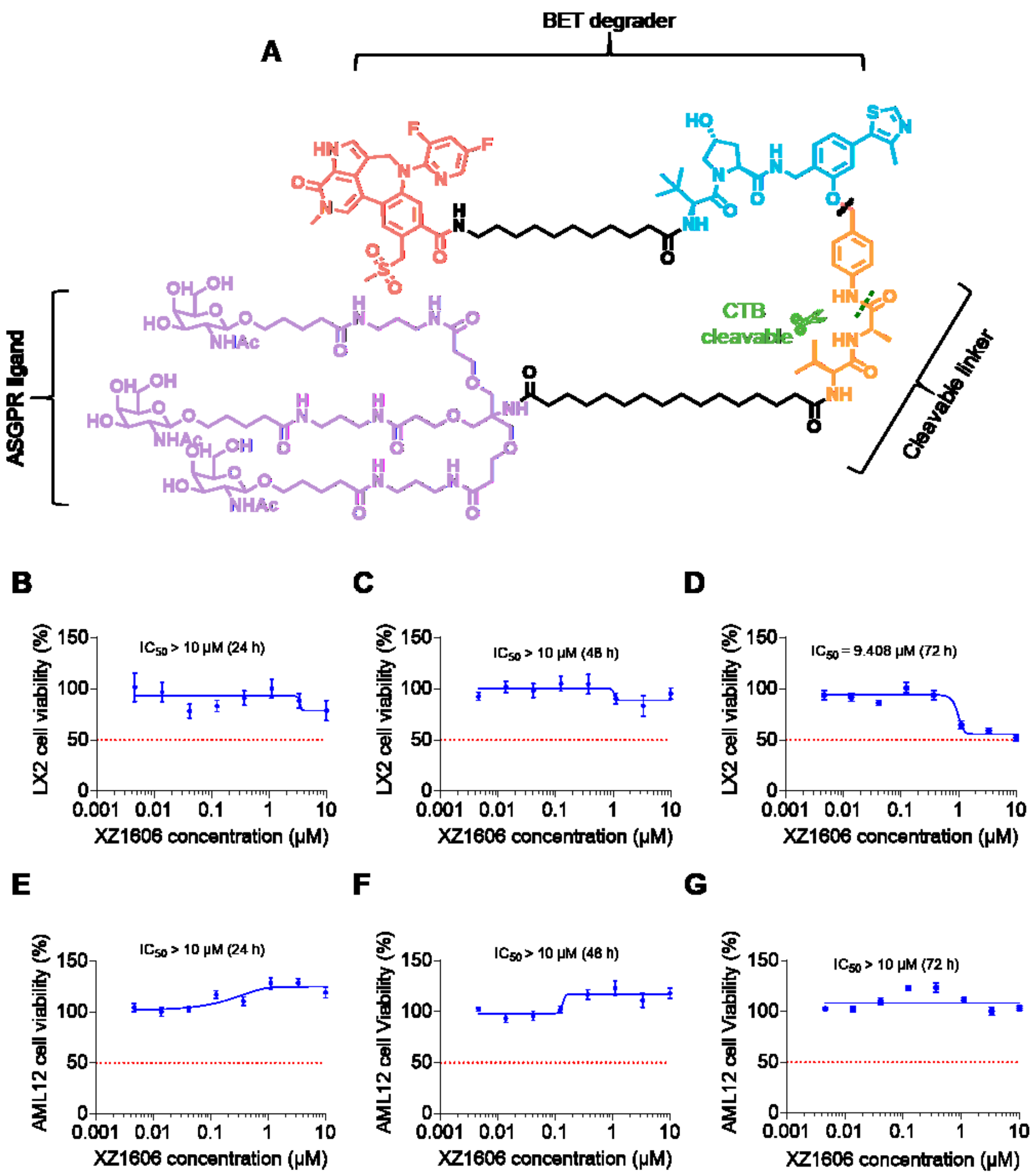

Figure S1. The design and cell viability effects of the Liver-Targeting Chimera (LIVTAC) XZ1606. **(A)** The chemical structure of XZ1606. LIVTAC consists of three key components: an ASGPR-targeting tri-GalNAc moiety for liver-specific delivery, a cathepsin B-cleavable linker for controlled release, and a BET degrader (GNE-987 scaffold) that promotes BRD4 degradation via the ubiquitin–proteasome pathway. **(B-D)** The viability of hepatic stellate LX-2 cells treated with increasing concentrations (0–10 μM) of XZ1606 over different time points. At 24 and 48 h (**B, C**), XZ1606 did not significantly impact cell viability, with IC_{50} values exceeding 10 μM. However, at 72 h (**D**), a reduction in LX-2 cell viability was observed, with an IC_{50} of 9.408 μM, indicating a delayed cytotoxic effect. **(E-G)** The effect of XZ1606 on AML12 hepatocytes under the same conditions. Across all time points (24, 48, and 72 h), no significant cytotoxicity was observed, with IC_{50} values consistently above 10 μM.

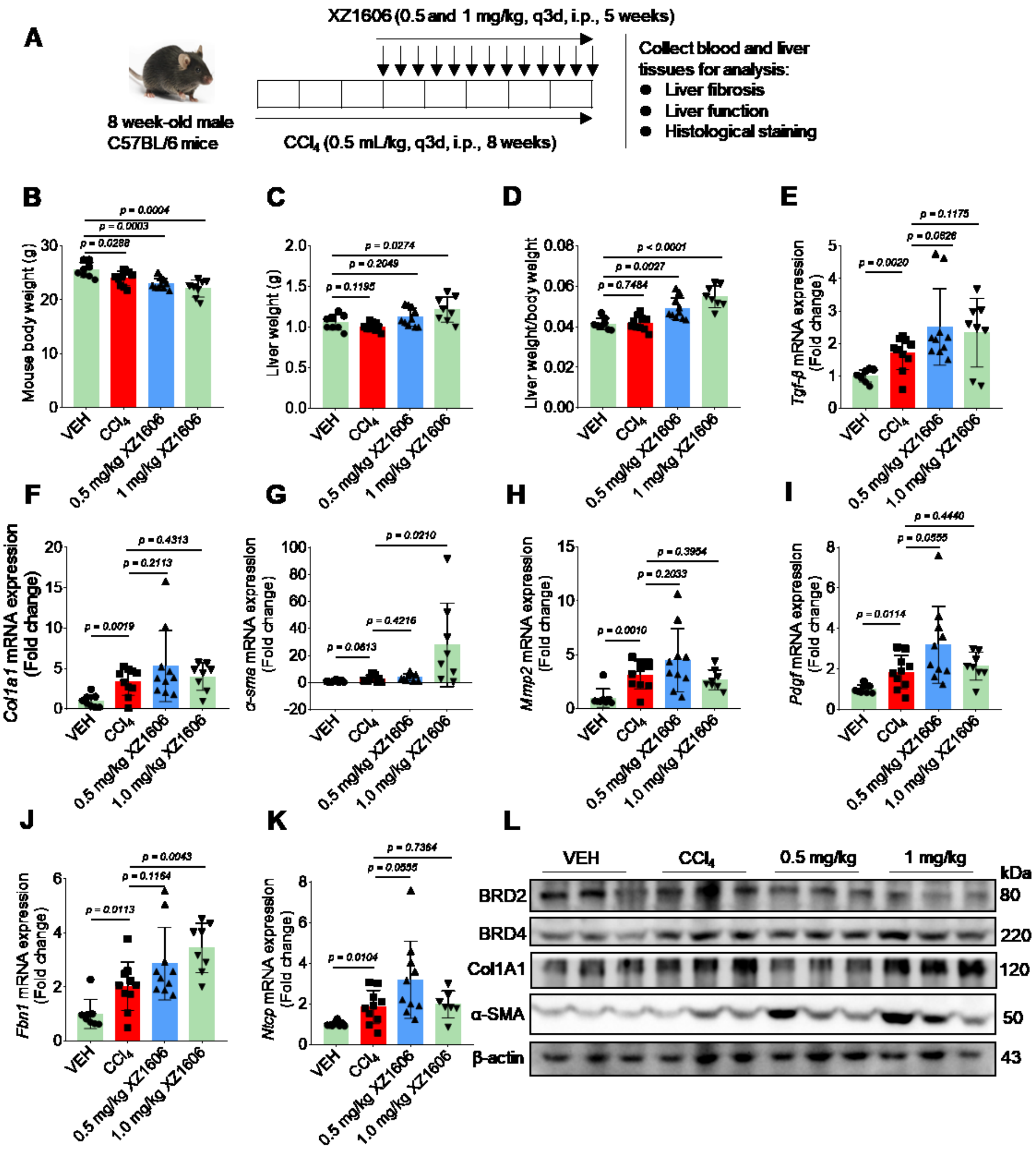

Figure S2. Experimental design and dose optimization for the CCl₄-induced liver fibrosis mouse model treated with XZ1606. (A) Experimental design: C57BL/6 mice were administered CCl_4 (0.5 mL/kg) for 8 weeks to induce liver fibrosis. Following this, the mice were treated with XZ1606 at doses of 0.5 mg/kg and 1 mg/kg, every third day for 5 weeks. The four experimental groups were: Vehicle (n = 8), CCl_4 + Vehicle (n = 10), CCl_4 + XZ1606 0.5 mg/kg (n = 10), and CCl_4 + XZ1606 1 mg/kg (n = 8). **(B)** Body weight of mice across the four experimental groups during the 5-week treatment period. No significant differences were observed between the groups. **(C)** Liver weights of mice from each group at the end of the treatment. **(D)** Liver weight to body weight ratio, serving as an index of liver

enlargement, showed no significant reduction in the XZ1606 groups compared to the CCl_4 + Vehicle group. **(E-K)** Quantitative PCR analysis of fibrosis-related gene expression in liver tissues: **(E)** *Tgfb*, **(F)** *Col1a1*, **(G)** *α-sma*, **(H)** *Mmp2*, **(I)** *Pdgf*, **(J)** *Fbn1*, and **(K)** *Ntcp*. Data are presented as fold change relative to the Vehicle group and normalized to GAPDH expression. No significant changes in mRNA expression were observed in XZ1606 treatment groups. **(L)** Western blot analysis assessing the protein expression levels of collagen I (COL1A1), smooth muscle actin (α-SMA), bromodomain-containing protein 2 (BRD2), and BRD4 in liver tissues. No significant differences in protein levels were found between the CCl_4 + XZ1606 and CCl_4 + Vehicle groups, indicating minimal effect of XZ1606 on fibrosis markers. These results suggest that XZ1606 treatment at doses of 0.5 mg/kg and 1 mg/kg does not significantly impact liver fibrosis in the CCl_4-induced mouse model.

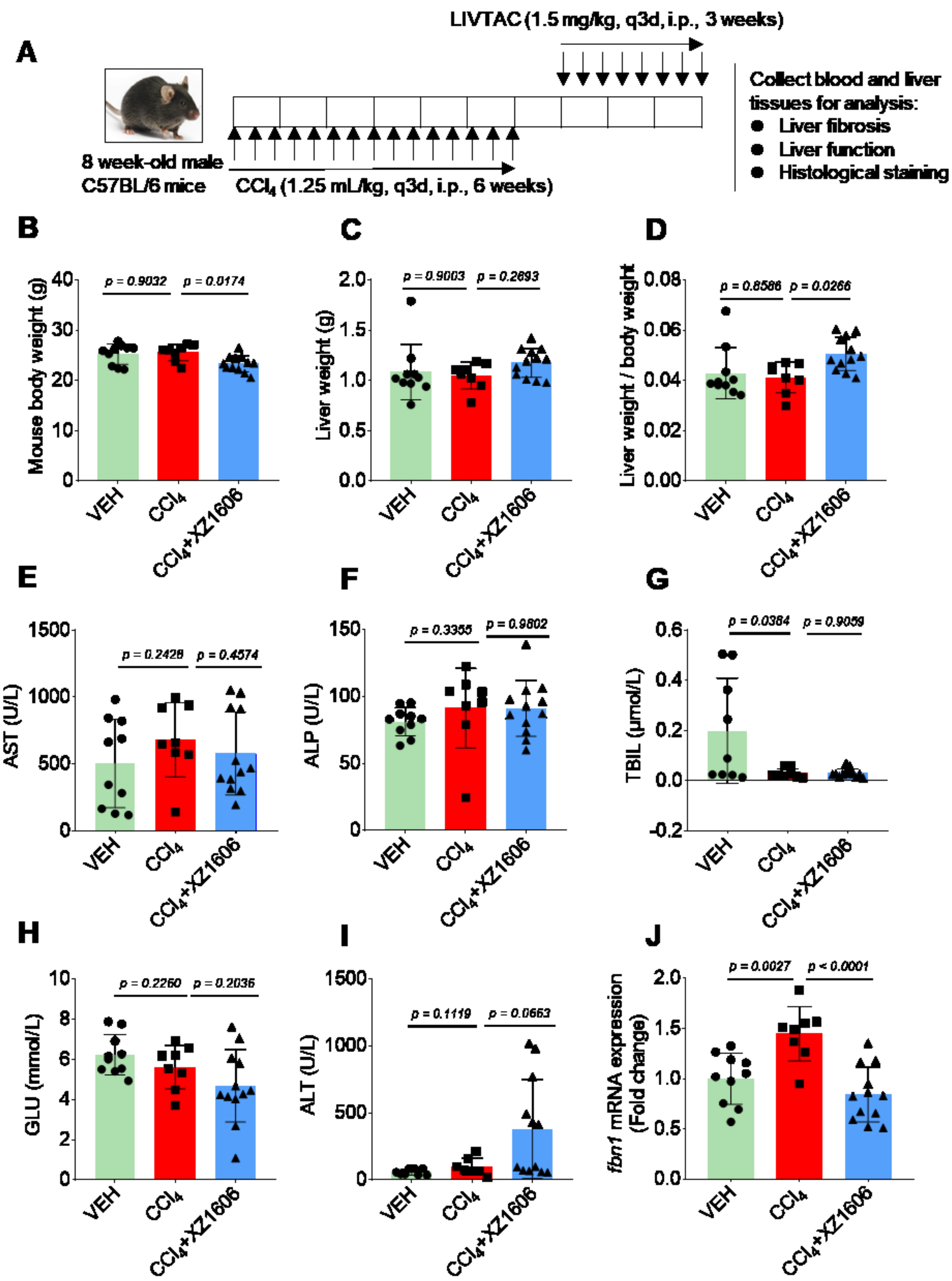

Figure S3. Experimental design and outcomes of the CCl_4-induced liver fibrosis mouse model treated with XZ1606. (A) Experimental setup: C57BL/6 mice were administered CCl_4 (1.25 mL/kg) for 6 weeks to induce liver fibrosis. Following this, the mice were treated with XZ1606 at a dose of 0.5 mg/kg, every third day for 3 weeks. The

three experimental groups were: Vehicle (n = 10), CCl_4 (n = 8), and CCl_4 + XZ1606 1.5 mg/kg (n = 12). **(B)** Body weight measurements of mice in each group over the 6-week treatment period. No significant differences were observed between the groups, indicating no marked systemic effects. **(C)** Liver weights of mice from each group at the end of the treatment period. No significant reduction in liver weight was observed in the XZ1606 treatment group compared to the CCl_4 + Vehicle group. **(D)** Liver weight to body weight ratio, an indicator of liver enlargement, showed no significant changes in the XZ1606 treatment groups. **(E)** Serum aspartate aminotransferase (AST) levels, a marker of liver injury, were measured. No significant changes in AST levels were observed among the groups. **(F)** Alkaline phosphatase (ALP) levels, another indicator of liver injury, did not show significant differences between treatment groups. **(G)** Total bilirubin (TBIL) levels, indicating liver function, showed no significant effects of XZ1606 treatment. **(H)** Glucose (GLU) levels were measured to assess metabolic changes, with no significant differences observed between the groups. **(I)** Serum alanine aminotransferase (ALT) levels, another liver function marker, were also not significantly affected by XZ1606 treatment. **(J)** Fibrinogen (*Fbn1*) mRNA expression was significantly reduced in the CCl_4 + XZ1606 group compared to the CCl_4 + Vehicle group, suggesting a potential effect on fibrosis markers.

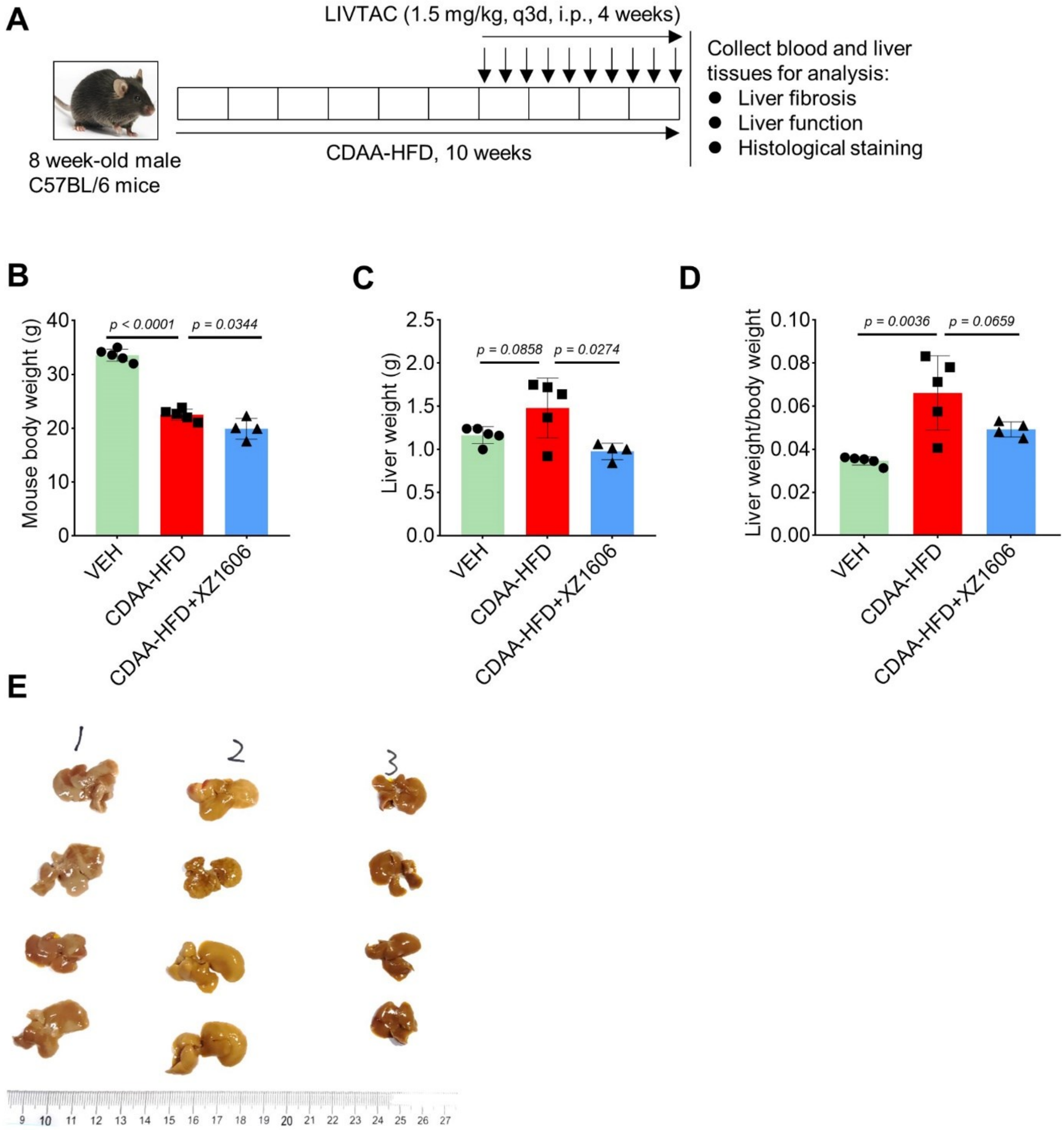

Figure S4. CDAA-HFD Model and XZ1606 Treatment Design. (A) Mice were fed a choline-deficient, L-amino acid-defined, high-fat diet (CDAA-HFD) for 10 weeks to induce liver fibrosis, followed by a 4-week treatment with XZ1606 (1.5 mg/kg, i.p., every 3 days). The three experimental groups were: Vehicle (n = 5), CDAA-HFD (n = 5), and CDAA-HFD + XZ1606 (n = 4). **(B)** Body weights of mice were recorded during the treatment period. **(C)** Liver weights were measured at the end of the study. (**D**) The ratio of liver weight to body weight was calculated as an index of hepatomegaly. **(E)** Representative liver images showing macroscopic changes across the groups. Data are presented as mean ± SEM. Statistical significance was determined by one-way ANOVA followed by Tukey's post-hoc test.
